# Blocking Tim-3 enhances the anti-tumor immunity of STING agonist ADU-S100 by unleashing CD4^+^ T cells through regulating type 2 conventional dendritic cells

**DOI:** 10.7150/thno.86792

**Published:** 2023-09-04

**Authors:** Jing Luo, Shuju Pang, Zhenzhen Hui, Hua Zhao, Shilei Xu, Wenwen Yu, Lili Yang, Qian Sun, Xishan Hao, Feng Wei, Jian Wang, Xiubao Ren

**Affiliations:** 1 Tianjin Medical University Cancer Institute and Hospital, National Clinical Research Center for Cancer, Tianjin, 300060, China.; 2 Tianjin's Clinical Research Center for Cancer, Tianjin, 300060, China.; 3 Key Laboratory of Cancer Immunology and Biotherapy, Tianjin, 300060, China.; 4 Department of Immunology, Tianjin Medical University Cancer Institute and Hospital, Tianjin, 300060, China.; 5 Department of Biotherapy, Tianjin Medical University Cancer Institute and Hospital, Tianjin, 300060, China.; 6 Laboratory of Cancer Cell Biology, Tianjin Medical University Cancer Institute and Hospital, Tianjin 300060, China.; 7 Haihe Laboratory of Cell Ecosystem, Tianjin 300060, China.

**Keywords:** STING, ADU-S100, Tim-3, DC, Cancer immunotherapy

## Abstract

**Rationale:** An immunosuppressive tumor microenvironment (TME) is a major obstacle in tumor immunotherapy. Stimulator of interferon genes (STING) agonists trigger an inflammatory innate immune response to potentially overcome tumor immunosuppression. While STING agonists may hold promise as potential cancer therapy agents, tumor resistance to STING monotherapy has emerged in clinical trials, and the mechanisms remain unclear.

**Methods:** The *in vivo* anti-tumor immunity of STING agonist ADU-S100 (S100), plus anti-T cell immunoglobulin and mucin-domain containing-3 antibody (αTim-3) were measured using murine tumor models. Tumor-specific T cell activation and alterations in the TME were detected using flow cytometry. The maturation and function of dendritic cells (DC) were also measured using flow cytometry, and the importance of CD4^+^ T cells in combination therapy was measured by blocking antibodies. Additionally, the effect of S100 on CD4^+^ T was verified via *in vitro* assays. Lastly, the impact of conventional dendritic cells (cDC) 2 with a high expression of Tim-3 on survival or therapeutic outcomes was further evaluated in human tumor samples.

**Results:** S100 boosted CD8^+^ T by activating cDC1 but failed to initiate cDC2. Mechanistically, the administration of S100 results in an upregulation of Tim-3 expressed in cDC2 (Tim-3^+^cDC2) in both mice and humans, which is immunosuppressive. Tim-3^+^cDC2 restrained CD4^+^ T and attenuated the CD4^+^ T-driven anti-tumor response. Combining S100 with αTim-3 effectively promoted cDC2 maturation and antigen presentation, releasing CD4^+^ T cells, thus reducing tumor burden while prolonging survival. Furthermore, high percentages of Tim-3^+^cDC2 in the human TME predicted poor prognosis, whereas the abundance of Tim-3^+^cDC2 may act as a biomarker for CD4^+^ T quality and a contributing indicator for responsiveness to immunotherapy.

**Conclusion:** This research demonstrated that blocking Tim-3 could enhance the anti-tumor immunity of STING agonist ADU-S100 by releasing CD4^+^ T cells through regulating cDC2. It also revealed an intrinsic barrier to ADU-S100 monotherapy, besides providing a combinatorial strategy for overcoming immunosuppression in tumors.

## Background

The innate immune pathway cyclic guanosine monophosphate-adenosine monophosphate (cGAMP) synthase (cGAS)-stimulator of interferon genes (STING) plays a critical role in immune response to infection or cancer 1. STING was first identified as an important player in DNA-mediated innate immunity. It is localized within the endoplasmic reticulum or mitochondria-associated membranes, and is widely expressed in both immune and non-immune cells 2. When STING is activated by cGAMP under physiological conditions, it translocates to the Golgi and activates tumor necrosis factor receptor-associated factor (TRAF) family member-associated NF-kappa-B activator (TANK)-binding kinase 1 (TBK1) 2, 3. The STING-TBK1 complex then phosphorylates the interferon regulatory factor 3 (IRF3) transcription factor. IRF3 enters the nucleus, stimulating the production of type I interferons (IFN-I) and proinflammatory cytokines, and subsequently develops an immuno-supportive microenvironment 4, 5. Over the past decade, STING has been demonstrated as an encouraging target in antitumor immunity 6. Indeed, numerous STING agonists have been developed and assessed for antitumor immunity, with several candidates undergoing clinical trials 7. Despite the encouraging therapeutic outcomes in murine models, current STING agonists did not achieve satisfactory results in the clinical evaluations, while multiple agonists failed to show effects altogether in human patients 8, 9. ADU-S100 (S100) is a novel synthetic agent that activates the STING axis both *in vivo* and *in vitro* 7. S100 has comparable activities toward mouse and human STING, making it a promising therapeutic agent 9; however, clinical evaluations of S100 have not been successful 10, with some terminated preemptively 11. Accordingly, it was assumed that the efficacy of this STING agonist as a single therapeutic agent was not sufficient to progress to further clinical analyses. Additionally, studies indicated that S100 may diminish tumor-specific T cell responses, negatively affecting immunity 12; although, the precise mechanisms contributing to the lack of S100 monotherapy efficacy remain poorly defined. Recently, combinations of STING with other cancer drugs have become an appealing strategy; yet a clinical trial (NCT 03172936) combining ADU-S100 with anti-programmed death-1 (PD-1) also yielded disappointing responses 8. Accordingly, these results support further examination of the precise antitumor mechanisms of STING agonists.

Numerous studies have been conducted on STING agonists, and many of them focus on the cDC1-mediated CD8^+^T cell response, the role of cDC2 still needs to be clarified. However, our research revealed that cDC2-mediated CD4^+^T cell response is required to improve the efficacy of STING agonists. In our previous study, we found that anti-T cell immunoglobulin and mucin-domain containing-3 antibody (αTim-3), another immune checkpoint inhibitor with poor clinical response in monotherapy 13, could potentiate the antitumor response of STING agonist S100. In a further investigation, we revealed that S100 can activate type 1 conventional DCs (cDC1) but fail to initiate cDC2, further it upregulated the expression of Tim-3 in cDC2, which have immunosuppressive properties on CD4^+^ T. This may partly explain the poor response of S100 mono- or combination therapy in clinical trials. Tim-3 was identified as a new molecule belonging to a family of immune-checkpoint receptors and is considered an important emerging immune checkpoint target 14, 15. The expression of Tim-3 on CD8^+^ T cells is considered a sign of T cell dysfunction 15; however, Tim-3 is also expressed on several other types of immune cells 16, 17. It was reported that there is singular importance of blocking Tim-3 on DCs, but not on T or other immune cells, promoting strong anti-tumor immunity 18 and making Tim-3 on DCs a novel checkpoint for immunotherapy. The precise mechanisms underlying the tumor-promoting activity of Tim-3 expressed on DCs, however, are not well revealed. In our research, Tim-3 expressed on cDC2 has an inhibitory effect on CD4^+^ T, providing a partial understanding of Tim-3's role on DCs.

In addition to STING activation, the present study found that S100 monotherapy could upregulate the expression of Tim-3 in cDC2, subsequently diminishing the CD4^+^ T cell-mediated anti-tumor immunity; therefore, this negative impact could be relieved by αTim-3. The immunosuppressive regulation of Tim3^+^cDC2 on CD4^+^ T was identified as well. Notably, Tim-3^+^cDC2 showed that minimal impacts on CD8^+^ T cells do not mean that CD8^+^ T is unimportant. Rather, CD8^+^ T subsets are critical to the anti-tumor immunity of S100; however, they are not the major target rescued by αTim-3. Furthermore, an investigation of tumor samples of patients conferred a negative correlation of Tim3^+^cDC2 co-signatures with survival. Thus, blocking Tim-3 of cDC2 cells in STING pathway offers a promising strategy for safe and efficacious cancer immunotherapy.

## Materials and Methods

### Animal studies

#### Mice and treatment

**Figure 1:**
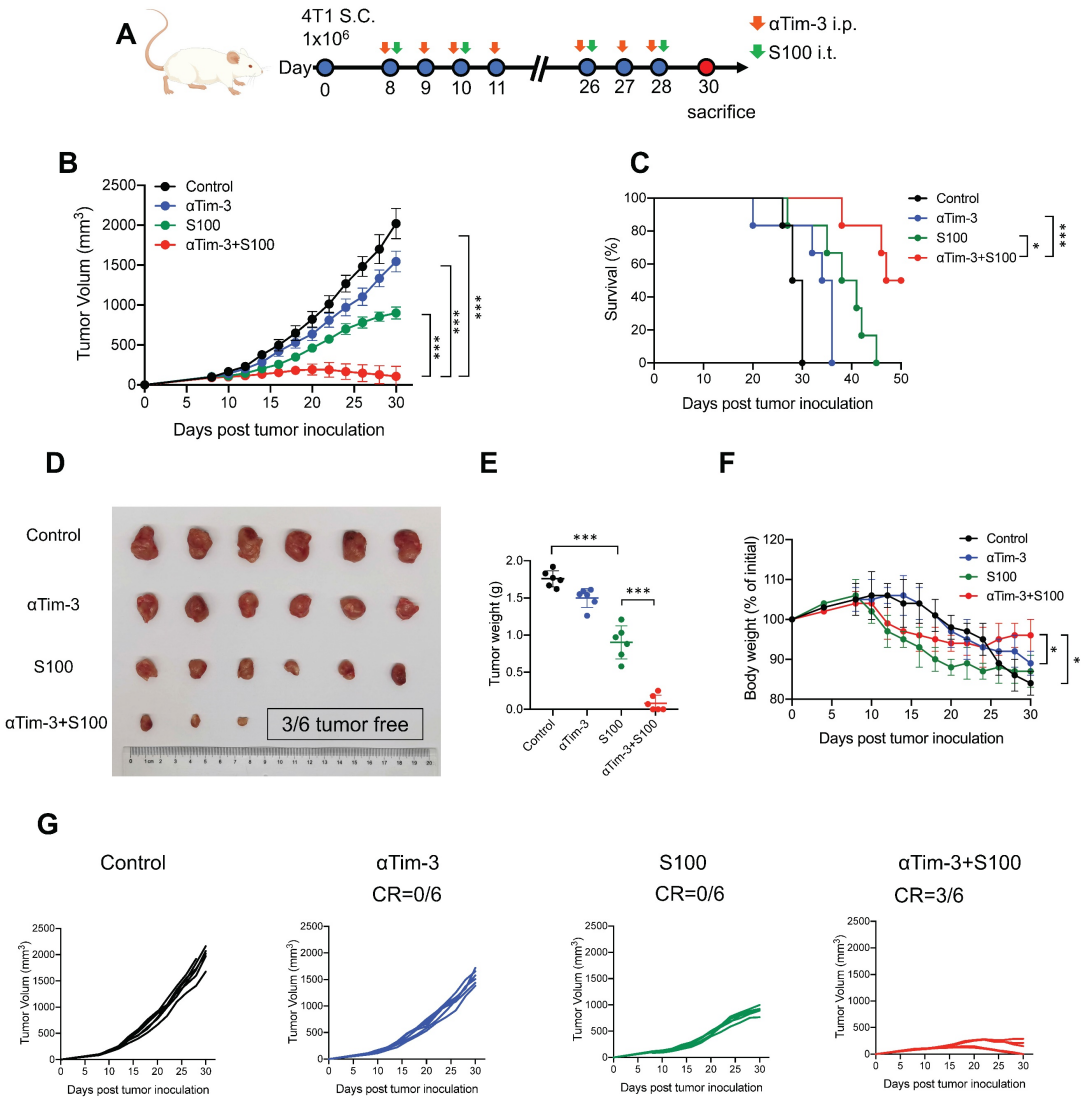
** αTim-3 significantly increased the antitumor response of S100 in 4T1 tumor model.** (**A**) Workflow for the 4T1 breast tumor model and treatment with S100 + αTim-3. (**B**) Tumor volume, (**C**) survival, (**D**) representative images of tumors, (**E**) tumor weights, (**F**) body weight changes of mice bearing 4T1 tumors treated with S100 or αTim-3 monotherapies, as well as the combination therapy compared with control treatment (n = 6 mice per group). (**G**) Tumor growth curves of individual mice in different groups. S100, ADU-S100; αTim-3, anti-Tim-3; S.C., subcutaneous injection; i.p., intraperitoneal injection; i.t., intertumoral injection; CR, complete response. Data are presented as means ± SD. *p < 0.05; ***p < 0.01. Unpaired two-tailed Student's t-test.

Six- to eight-week-old WT female BALB/c and C57BL/6 mice were purchased from SPF Biotechnology (Beijing). On day 0, 1 × 10^6^ 4T1 cells/100 μL PBS were injected into the right groin of the BALB/c mice and 5× 10^5^ B16F1 cells/100 μL PBS or 5× 10^5^ B16F1-OVA cells/100 μL PBS were injected into the right groin of the C57BL/6 mice. Tumor growth was monitored daily and measured every 1-2 days. Tumor volume was determined as length (mm) × width (mm^2^) × 0.5. When tumor volume reached approximately 80-100 mm^3^, the mice were randomly divided into treatment groups. For the S100 treatment, tumor bearing mice were treated intratumorally once with saline and 50 μg S100 every 2 day during the first week. Then, doses of S100 were decreased to 30 μg, while the administration frequency remained unchanged. For αTim-3 treatment, tumor-bearing mice were treated intraperitoneally with 250 μg αTim-3 monoclonal antibody daily. Mice in the control group were given IgG2a isotype. All treatment details are provided in Figure 1A and Figure S1A. Mice were sacrificed when tumor volume reached 2000 mm^3^. In the rechallenge experiments, tumor-free survivors were rechallenged with tumor cells (1 × 10^6^ 4T1 cells or 5× 10^5^ B16F1 cells) on the same site several weeks after the primary tumor was totally cured. Normal WT mice were used as controls. OT-I and OT-II mice were from Cyagen Bioscience (Santa Clara, CA, USA). All mice were used according to protocols approved by the Animal Ethical and Welfare Committee of Tianjin Medical University Cancer Institute and Hospital and maintained in pathogen-free conditions within a barrier facility.

#### Cells

4T1 was cultured in complete medium (medium with 10% fetal bovine serum, 1% penicillin-streptomycin) RPMI-1640, and B16F1 was cultured in complete medium DMED. All cell lines were obtained from ATCC and tested negative for mycoplasma contamination. To obtain Bone marrow derived dendritic cells (BMDCs) bone marrow cells were flushed out with PBS, cultured in complete medium RPMI-1640, and then supplemented with 10 ng/mL IL-4 and 20 ng/mL GM-CSF. The medium was half replenished every three days with total dose of IL-4 and GM-CSF. Immature DCs which were suspended and loosely adherent were collected on day 6, and flow cytometry was used to detect the expression of CD11c (regularly 50-70% CD11c^+^). CD11c magnetic beads were used to purify BMDCs. CD4^+^ T/CD8^+^ T cells and natural killer (NK) cells were isolated from spleens using magnetic beads, then cultured in complete medium RPMI-1640 with 20 ng/mL IL-2. Bone marrow-derived macrophages (BMDM) were generated by culturing the bone marrow cells that were flushed from the femurs of BALB/c naïve mice, cultured in complete medium RPMI-1640, and then supplemented with 25 ng/mL M-CSF. Mature BMDM were collected on Day 7. For human DC generation, umbilical cord blood (UCB) was used as a source of hematopoietic stem cells (HSCs). To obtain HSCs, lymphocytes were purified by Ficoll from UCB, then CD34 magnetic beads were used to isolate CD34^+^ HSCs. Cells were cultured in complete CellGenix GMP DC serum-free medium, and then supplemented with 10 ng/mL IL-4, 20 ng/mL GM-CSF and 25 ng/mL Flt3 ligand. The medium was half replaced every three days with the total dose of cytokines. Mature DCs were collected on day 7.

#### Pathological response of major organs in mice to the treatment

When the treatments (S100 and αTim-3 monotherapies, or their combination) were completed, major organs of mice were collected from each cohort, hematoxylin and eosin (HE) staining of slides was performed, while lymphocytic infiltration, necrosis, and hemorrhage foci were observed independently by two pathologists.

#### Flow cytometry

Tumor tissues or tumor-draining lymph nodes (tDLNs) were harvested from mice, then minced and digested in a mixture of 160 μg/mL collagenase Ⅳ and 50 μg/mL DNase Ⅰ in RPMI 1640 media at 37 ℃ for 30-40 minutes with agitation, before being strained through a 70 μm filter. For staining of single cell suspensions, all incubations were performed on ice. Cells were first incubated with Zombie NIR (1/500), diluted in PBS for 30 minutes to distinguish dead and living cells, then washed twice, incubated with a mixture of antibodies in fluorescence-activated cell sorting (FACS) buffer (2% fetal bovine serum in PBS) for 30 minutes, washed twice again, and suspended in staining buffer. Intracellular staining for chemokines was performed in Perm/Wash buffer, followed by a washing step, and suspension in FACS buffer. T-helper (Th) 1/2 cells were quantified by intracellular cytokine staining for IFN-γ (Th1) and IL-4 (Th2), then, the Th1/Th2 ratio was calculated as the percentage of Th1 cells divided by the percentage of Th2 cells 19. Intranuclear staining of Foxp3 using a Foxp3/Transcription Staining Buffer Set according to the manufacturer's instructions. For the tetramer staining, lymphocytes isolated from spleens were stained with tetramer for 60 minutes at 4 °C, shield from light. Cells were washed twice and then followed by staining with other surface markers. Data were collected using BD LSR Fortessa or Beckman CytoFLEX flow cytometer and analyzed in FlowJo version 9. Immune population gating strategies are provided in Figure S6.

#### Western blotting

To assess the activation of the cGAS-STING pathway, tumor tissues from different treatment groups were isolated and ground with liquid nitrogen. Total cell lysis buffer 1.1 × SDS with phosphatase inhibitor cocktail was used to generate whole-cell proteins. The quantity and quality of proteins was then confirmed using the NanoDrop (DeNovix DS-11) system. Purified proteins were electrophoresed in 10% Tris-Glycine gels, transferred to polyvinylidene fluoride membranes blocked using 5% bovine serum albumin solution, and blotted with the corresponding primary, secondary antibodies and horseradish peroxidase-conjugated IgG Ab for 2 hours (h). Membrane-bound complexes were detected using Image Studio.

#### *In vitro* cytotoxic T-lymphocyte assay

Spleens were harvested from OT-I mice and then minced and strained through a 70-μm filter to obtain single-cell suspensions. CD8^+^ T cells were purified using magnetic beads according to the manufacturer's protocol. CD8^+^ T cells were mixed with tumor cells (B16F1-OVA) in a 96-well U-bottom plate (CD8^+^ T: B16-OVA = 20:1). The plate was then briefly centrifuged at 1,500 rpm and then incubated at 37°C for 4 h. After completing the incubation, the plate was centrifuged again at 1,500 rpm. Then, 50 µL of the supernatant was collected and added to an opaque 96-well flat-bottom plate, and tumor cell survival was measured according to the manufacturer's protocol.

#### Enzyme-linked immunosorbent assay (ELISA)

For *in vivo* detection, blood samples were collected from treated mice. The serum was isolated and diluted in PBS based on the range of ELISA detection. For *in vitro* detection, the supernatants of the co-culture system were collected and stored at -80 ℃. ELISA kits were used to determine the levels of cytokines, transaminase, and chemokines. The protocols were performed according to the manufacturer's instructions.

#### Enzyme-linked immunosorbent spot (ELISpot)

For purification of CD4^+^ T or CD8^+^ T cells, the spleens were harvested from mice bearing 4T1 tumors after 24 h of intratumoral treatment with control, S100, and S100 plus αTim-3. Single-cell suspensions were prepared using magnetic beads according to the manufacturer's protocol. The sorted CD4^+^ T or CD8^+^ T cells were cocultured with BMDCs and tumor cell debris in a 96-well plate for 24 h. IFN-γ-producing T cell numbers were visualized using anti-mouse IFN-γ ELISpot assay (Dakewe) according to the manufacturer's protocol. The spots were counted and analyzed using the AID ELISpot plate reader software (Autoimmun Diagnostika).

#### Real-time PCR

CD45^+^ immune cells were isolated from single cell suspension of tumor tissues using magnetic beads. Total RNA was extracted from tumor tissues using TRIzol reagent, according to the manufacturer's instruction. Random primer and SuperScript III reverse transcriptase were used to synthesize cDNA, and then quantitative RT-PCR was performed using 2X SG Fast qPCR Master Mix (Low Rox) according to the manufacturer's instructions. The DNA primers used were as follows: mouse IFN-β F, ATGAGTGGTGGTTGCAGGC; mouse IFN-β R, TGACCTTTCAAATGCAGTAGATTCA; mouse CXCL-9 F, TCCTTTTGGGCATCATCTTCC; and mouse CXCL-9 R, TTTGTAGTGGATCGTGCCTCG.

#### *In vivo* immune cell depletion and blockade using antibodies

For the *in vivo* immune cell depletion experiment, corresponding *InVivo*Mabs were used. For CD4^+^ or CD8^+^ T subset depletion, mice were given 250 μg anti-CD8a or anti-CD4 antibodies via intraperitoneal injection on day 3 after tumor inoculation, and this dosage was maintained every 3 days. NK cells were depleted *in vivo* by i.p. injection with 30 μL anti-Asialo-GM1 antibody on day 0, 1, and 3 after tumor inoculation. NK depletion was maintained by i.p. injection of 20 μL anti-Asialo-GM1 antibody on days 7, 10, and 13 20. The depletion of mouse macrophages was achieved by clodronate-liposome intravenous injection 3 days after tumor inoculation, including 100 µL of clodronate liposome per 10 g of body weight. The first injection was given once in 24 h, then intravenous injections were delivered every 2 days to maintain the depletion 21.

#### Induction high expression of Tim-3 on cDC2s by S100

Mature BMDCs were generated as described above and isolated using CD11c magnetic beads. The purified BMDCs were co-cultured with different concentrations of S100 (12.5 to 200 μM for 6, 12, 24, 36, and 48 h). Then, Tim-3 expression was detected via flow cytometry. Considering that S100 has minimal effect on cDC1, CD103^+^cDC1 was not removed. The lowest S100 concentration that could induce Tim-3^+^cDC2 was selected (25 μM).

#### T cell proliferation assay

Spleens from naive mice were examined using CD4 or CD8 magnetic beads to harvest T cells. Then, CD8^+^ or CD4^+^ T cells were labeled with CFSE according to the manufacturer's instructions. Tim-3^+^ cDC2 was induced by S100 at 25 μM. The Tim-3^-^ cDC2 was a normal cDC2 isolated from mature BMDCs. The mixture including T cells and Tim-3^+^ cDC2/ Tim-3^-^ cDC2 with or without αTim-3 was then incubated for 3-5 days. The medium containing S100 was replaced every 2 days with the total dose of S100. Flow cytometry was performed to measure the ratio of CFSE-labeled cells.

#### T cell migration assay

CD8^+^ or CD4^+^ T cells were harvested from mouse spleens, and Tim-3^+^cDC2 was induced by S100 as described above. To test the effects on the migration ability of T cells from Tim-3^+^ cDC2, 1 × 10^5^ CD8^+^ or CD4^+^ T cells were resuspended in 200 μL of serum-free medium and plated in the upper chamber of a 24-well transwell plate. A total of 600 μL medium containing 1 × 10^4^ Tim-3^+^ cDC2/Tim-3^-^ cDC2 with or without αTim-3 was placed in the lower chamber as a chemoattractant, while 1 × 10^3^ tumor cells (4T1) were also added. Following 12-24 h of incubation, the migratory T cells in the lower chamber were counted using flow cytometry.

#### Multiplex cytokine and chemokine array

Tim-3^+^cDC2 was induced by S100 as described above. The supernatant of the 3-4-day co-culture system, including tumor cell (4T1) and CD4^+^ T cells, and Tim-3^+^cDC2/Tim-3^-^cDC2 and αTim-3 were analyzed for cytokine/chemokine concentrations using BD cytometric bead array mouse Th1/Th2/Th17 cytokine kits according to the manufacturer's instructions.

#### *In vitro* priming assay

CD4^+^ T cells were isolated from the spleens of OT-II mice (6-8 weeks old) using magnetic beads. Tim-3^+^cDC2 was induced by S100 as described above. Then, CD4^+^ T cells from OT-II mice were mixed with Tim-3^+^cDC2/Tim-3^-^cDC2 and αTim-3 and incubated with 0.1 μM ovalbumin peptide (OVA_323-339_) 22 (CD4^+^ T cells: Tim-3^+^cDC2/Tim-3^-^cDC2 = 2:1) for 24 h. The cytotoxicity of CD4^+^ T cells was analyzed using flow cytometry.

#### Adoptive transfer of immune cells

Immune cells were generated as described above and induced with 25 μM of S100 for 24 h, and then Tim-3 expression was detected using flow cytometry. A total of 2 × 10^6^ immune cells with high or low Tim-3 expression were intravenously injected into 4T1 tumor-bearing (tumor size 80-100 mm^3^) BALB/c mice a day before S100+αTim-3 treatment. After 48 h, the mice were sacrificed, and tumor tissues were obtained.

#### RNA-sequencing (RNA-seq)

Tim-3^+^cDC2 and Tim-3^-^cDC2 were induced by S100 as described above. TRIzol was used to extract total RNA from the samples, and the RNA concentration was evaluated using a NanoDrop 2000 spectrophotometer. The Illumina HiSeq platform was used to perform the RNA-seq assay. The DEseq2 package was applied to indicate differentially expressed genes (DEGs) between samples based on the following screening criteria: p < 0.05 and fold change > 1.2. Gene Ontology (GO) analyses were performed in the clusterProfiler package to reveal the potential functions of DEGs. The GO analysis identified terms in the categories biological process (BP), cellular component (CC), and molecular function (MF), and terms with p < 0.05 were considered significantly enriched.

### Human studies

#### Patients and design

Fifty-eight (58) patients with resectable non-small cell lung cancer (NSCLC) who received two cycles of neoadjuvant therapy— neoadjuvant chemotherapy (NAC) or neoadjuvant pembrolizumab and chemotherapy (NAPC)—were recruited in this study (30 patients received NAC, while 28 received NAPC). The study was approved by the Ethics Committee of Tianjin Cancer Institute and Hospital, and all patients enrolled in the study provided written informed consent. Patients were given NAC: paclitaxel 175mg·m^-2^ + carboplatin (area under curve 5; 5 mg/mL/min) for squamous cell carcinoma, and pemetrexed 500 mg/m^2^ + carboplatin for adenocarcinoma, with or without neoadjuvant immunotherapy pembrolizumab: 200 mg for two cycles. After finishing the neoadjuvant therapy, surgical resection was performed. Clinicopathological characteristics are provided in Table 1. Follow-ups on the patients were conducted from January 1, 2014 until October 10, 2022, with a median follow-up time of 97 and 35 months for NAC and NAPC patients, respectively. OS was calculated from the time of pathological diagnosis to the last follow-up or death. PFS was defined as the time from treatment initiation to disease progression, death, or the last follow-up. Tumor tissues from surgical resection were used by two pathologists to independently assess the pathological response to therapy. MPR was considered as ≤ 10% tumor cells to the total tissue area according to previously described methods 23, 24.

#### Multiplex immunohistochemistry (mIHC) and multispectral analysis

Formalin-fixed and paraffin-embedded (FFPE) tumor tissues were collected. mIHC staining was performed using a PerkinElmer Opal 7-color Technology Kit according to the manufacturer's instructions, with one panel of targets containing CD11c, CD1C, Tim-3, CD4, Foxp3, and DAPI. Details of antibodies are provided in the key resources table. Visualization and quantitation of each staining slide was achieved using TissueFAXS Spectra Systems and StrataQuest analysis (TissueGnostics), according to previously described methods 25. A multi-spectral image was scanned using a 20× objective lens, and 20 fields were selected at random in each slide. The quantification of spatial distribution between cells were performed using the dilate algorithm, defining the cell sociology for each selected area. Lastly, corresponding algorithms were developed according to analysis requirements (CD4^+^ T cell/Treg with Tim-3^-^cDC2/Tim-3^+^cDC2), and the unified algorithm and threshold for each channel were applied to all samples for standardizing the expression and fluorescence level of each marker. Based on previous studies 26, 27, Spatial distance analyses were performed for r = 30 μm, representing the proximity distance as the average number of cells distributed from the nuclear center of any reference cell.

#### T-cell receptor (TCR) sequencing and analysis

The tumor biopsies from 9 patients evaluated for single-cell TCR sequencing were collected from the 58 patients who received neoadjuvant therapy. TCR libraries were built by Chromium Single Cell V(D)J Enrichment Kit, Human T Cell (10x Genomics), Chromium Single Cell 5′ Library Construction Kit (10x Genomics), and i7 Multiplex Kit (10x Genomics) according to the manufacturers' instructions. Sequencing was performed on an Illumina HiSeq X Ten (Illumina, San Diego, CA, USA). TCR repertoire diversity was calculated using the Shannon index, which is the function of both the relative number of clonotypes present, and the relative abundance or distribution of each clonotype. The Shannon index was calculated according to Eq:



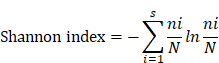



Where *ni* is the clonal size of the *i*th clonotype (that is, the number of copies of a specific clonotype), s is the number of different clonotypes, and *N* is the total number of TCR sequences analyzed. Clonality was measured as 1- (Shannon index)/ln (# of productive unique sequences) 28. TCR richness was defined as the number of unique clonotypes within a sample, and is a key component of diversity metrics (including Shannon entropy).

Details about the antibodies, chemicals, peptides, and proteins as well as commercial assays used in the present study are provided in Supplementary key resource table.

## Results

### αTim-3 significantly potentiates the antitumor response of S100 and augments antigen-specific T cell response

The efficacy of combination therapy using the mouse immune “cold” tumors, 4T1, and B16F1 cell lines were assessed. Mice bearing 4T1 murine breast cancer (sized 80-100 mm^3^) were treated intratumorally with S100, and intraperitoneally with αTim-3. The treatment schedule is provided in Figure 1A and Figure S1A. The results showed that although S100 monotherapy exhibited antitumor activity compared to the control and αTim-3 monotherapy, the therapeutic outcomes were not satisfactory; however, the combination therapy significantly delayed tumor growth, and prolonged survival compared with S100 or αTim-3 monotherapy (Figure 1B-E and Figure S1B-E). Notably, over half mice (3/6 in 4T1 models and 3/4 in B16F1 models) in the combination therapy group were tumor-free (Figure 1D, G and Figure S1D, G). Moreover, it was observed that the mice which received S100 monotherapy experienced weight loss (Figure 1F and Figure S1F). Subsequently, the toxicities of the two drugs and their combination were evaluated. Blood ELISA were performed to evaluate the secretion of inflammatory cytokine and hepatic transaminases. The results showed TNF-α was increased in the S100 monotherapy and combination groups (Figure S2A), while mice in the two groups also maintained higher levels of hepatic transaminases in blood (Figure S2A). Accordingly, it appeared that STING agonist S100 led to a certain degree of toxicity. Importantly, no lymphocytic infiltration and local necrosis was observed from the HE staining results, suggesting the drugs were not directly toxic to vital organs (Figure S2B). Overall, the anti-tumor immunity response was tolerable.

**Figure 2:**
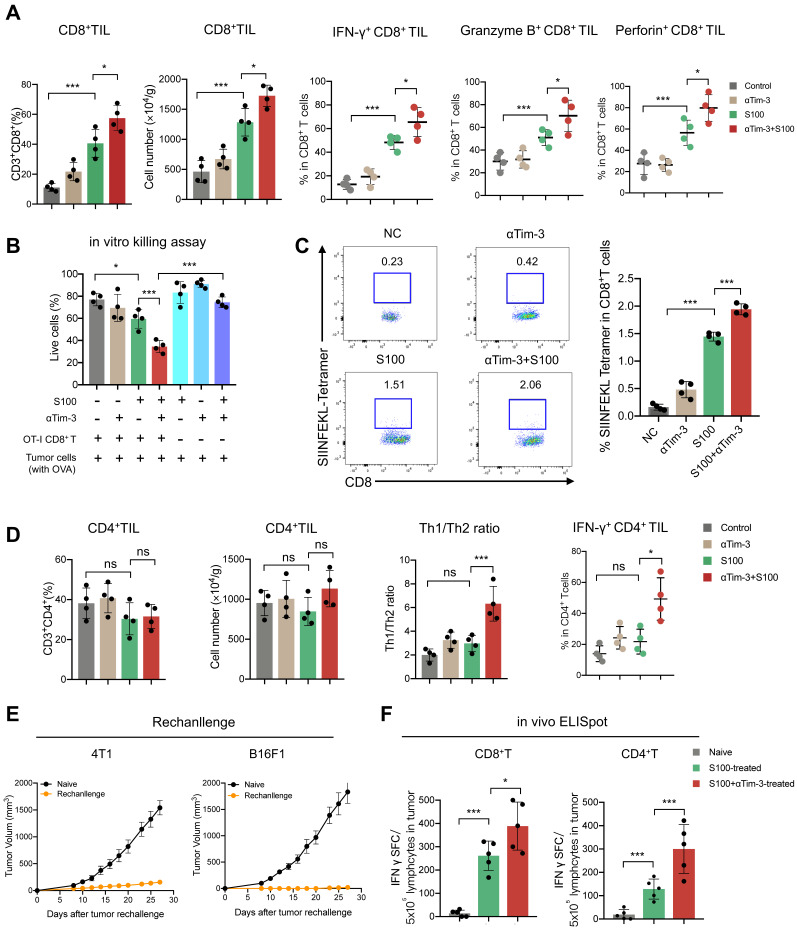
** S100 combined with αTim-3 stimulated T cell immune response.** (**A**) Quantification of tumor-infiltrating CD8^+^ T cells and the IFNγ^+^ CD8^+^ TILs, Granzyme B^+^ CD8^+^ TILs, and Perforin^+^ CD8^+^ TILs among CD8^+^ T cells of mice in different treatment groups. (**B**) Effect of the combination therapy on CD8^+^T cell-mediated lysis of tumor cells. CD8^+^T cell were isolated from spleen of OT-I mice, incubated with tumor cells (with ova) and with or without S100 or/and αTim-3. Viability of tumor cells were assessed by the lactate dehydrogenase cell death assay. (**C**) Representative image and quantification of tetramer^+^ CD8^+^ T cells in the spleen of mice bearing B16F1-OVA tumor in different treatment groups. (**D**) Quantification of tumor-infiltrating CD4^+^ T cells, the Th1/Th2 ratio as well as the IFN-γ^+^ CD4^+^ TILs among CD4^+^ T cells of mice in different treatment groups. (**E**) Tumor volume of mice rechallenged with 4T1 or B16F1 tumor cells compared with that of naive mice. Mice cured via combination therapy were rechallenged with 4T1 or B16F1 cells (n = 4 mice per group). Naive mice were also inoculated with 4T1 or B16F1 as a control (n = 4 mice per group). (F) Mice bearing 4T1 tumor treated with S100 or S100 plus αTim-3, CD8^+^, or CD4^+^ T cells isolated from mice spleens were cocultured with BMDCs and tumor cell debris in a 96-well plate for 24 h. IFN-γ-producing T cell numbers were evaluated through ELISpot assays. S100, ADU-S100; αTim-3, anti-Tim-3; TILs, tumor-infiltrating lymphocytes; BMDCs, bone marrow-derived DCs. Data are presented as means ± SD. *p < 0.05; ***p < 0.01; ns, not significant. Unpaired two-tailed Student's t-test.

The effects of combination therapy on the tumor microenvironment (TME) were also investigated. ELISA was performed to assess the levels of inflammatory cytokine IFN-γ and IFN-responsive chemokine (C-X-C motif) ligand 9 (CXCL)-9 and CXCL-10. Both IFN-γ and chemokines CXCL-9 and CXCL-10 increased in blood from mice in the S100 monotherapy and combination groups (Figure S3A). Moreover, the total proteins of mouse tumor tissues were extracted, and STING pathway activation was evaluated through western blotting (Figure S3B). Based on the ELISA and western blotting results, the STING pathway was more readily activated following S100 combined with αTim-3 than S100 monotherapy. To further differentiate the role of STING activation in host versus cancer cells, the expression levels of STING-downstream genes (*Ifnβ* and *Cxcl9*) in immune cells (CD45^+^) and cancer cells (CD45^-^) of the TME were quantified. The data showed that CD45^+^ cells exhibited higher level of STING activation over CD45^-^ populations, indicating that lymphocytes, rather than cancer or stromal cells, may be the primary targets for STING-mediated immunomodulation; thus, host STING is necessary for the combination therapy mediated anti-tumor immunity (Figure S3C). Furthermore, tumor-infiltrating lymphocytes (TILs) were measured using flow cytometry. Although compared to the control and αTim-3 monotherapy, S100 monotherapy increased the infiltration and proliferation of CD8^+^ T cells (Figure 2A) as well as CD8^+^ cytotoxic lymphocytes (CTL) in tumors, this was more significant in the combination group than S100 monotherapy. A marked increase of IFNγ^+^ CD8^+^ TILs, Granzyme B^+^ CD8^+^ TILs, and Perforin^+^ CD8^+^ TILs (Figure 2A) was observed in the combination group than in the S100 monotherapy. Furthermore, *in vitro* cytotoxic T cell killing assay was performed (Figure 2B) indicating that the killing ability of CTL was enhanced by S100 monotherapy and the combination therapy. Consistently, mice bearing B16F1-OVA tumors generated many more tetramer^+^CD8^+^T cells (Figure 2C) in the S100 monotherapy and combination groups, demonstrating an enhanced *in vivo* CTL killing ability. Based on the results described above, the anti-tumor response of CD8^+^T cells can be activated by S100 monotherapy and significantly promoted by the combination treatment of S100 and αTim-3. However, CD4^+^ T cells behaved differently, compared to the control and αTim-3 monotherapy, no obvious increase or activation of CD4^+^ T was observed in the S100 monotherapy. Although the CD4^+^ TILs also remained stable in the combination group, the proliferation of CD4^+^ T cells, the Th1/Th2 ratio as well as IFNγ^+^ CD4^+^ TILs also increased, indicating an increase of tumor-specific CD4^+^ T cells by the combination treatment (Figure 2D). By contrast, the reduction of tumor-infiltrating regulatory T cells (Treg; Figure S4) in the combination groups may partly explain the inconspicuous increase of CD4^+^ T cells in tumors. Further, other important immune cells were monitored in the combination treatment group. Tumor-infiltrating and tumor-associated macrophages (TAMs), including M1-like TAM, and M2-TAM, as well as myeloid-derived suppressor cells (MDSCs), B cells, and regulatory B cells (Breg), were found to be increased in the S100 monotherapy group; Treg and NK cells remained stable. All differences between the S100 monotherapy and combination groups were insignificant (Figure S4). Additionally, cured mice from the combination treatment were subsequently rechallenged with the same type of cancer cells. The results displayed a marked reduction of tumor recurrence in cured compared to naive mice (Figure 2E), suggesting a potential benefit for protective memory immunity after combination therapy. Besides, we also observed a significant increase of tumor-infiltrating tissue-resident memory T cells (Figure S5A) and effector memory T cells (Figure S5B) in both CD4^+^ and CD8^+^ T cell subsets from combination-treated mice, revealing memory CD8^+^ /CD4^+^ T cells were activated after combination therapy. To further investigate the memory immunity response, we examined whether the S100 plus αTim-3 affected the ability of memory T-cell priming. The IFN-γ-producing cell numbers were assessed using ELISpot. ELISpot results showed that both CD8^+^ and CD4^+^ T cells isolated from S100 plus αTim-3-treated mice exhibited a much better ability to initiate the production of IFN-γ than T cells isolated from S100-treated mice or naive mice when incubated with DCs and tumor cell debris. However, S100 monotherapy seemed to initiate memory CD8^+^- more easily than memory CD4^+^- T cell response (Figure 2F, S5). These results indicated that although S100 monotherapy has minor effect on CD4^+^T subsets, both antigen-specific CD4^+^ or CD8^+^ T cells were improved in S100 combined αTim-3 treatment.

### S100 is able to activate cDC1 but fails to initiate cDC2

The activation of professional antigen-presenting DCs is essential for antigen-specific T cell priming. The findings above show that antigen-specific T cells were significantly increased and activated in the combination treatment group.

**Figure 3:**
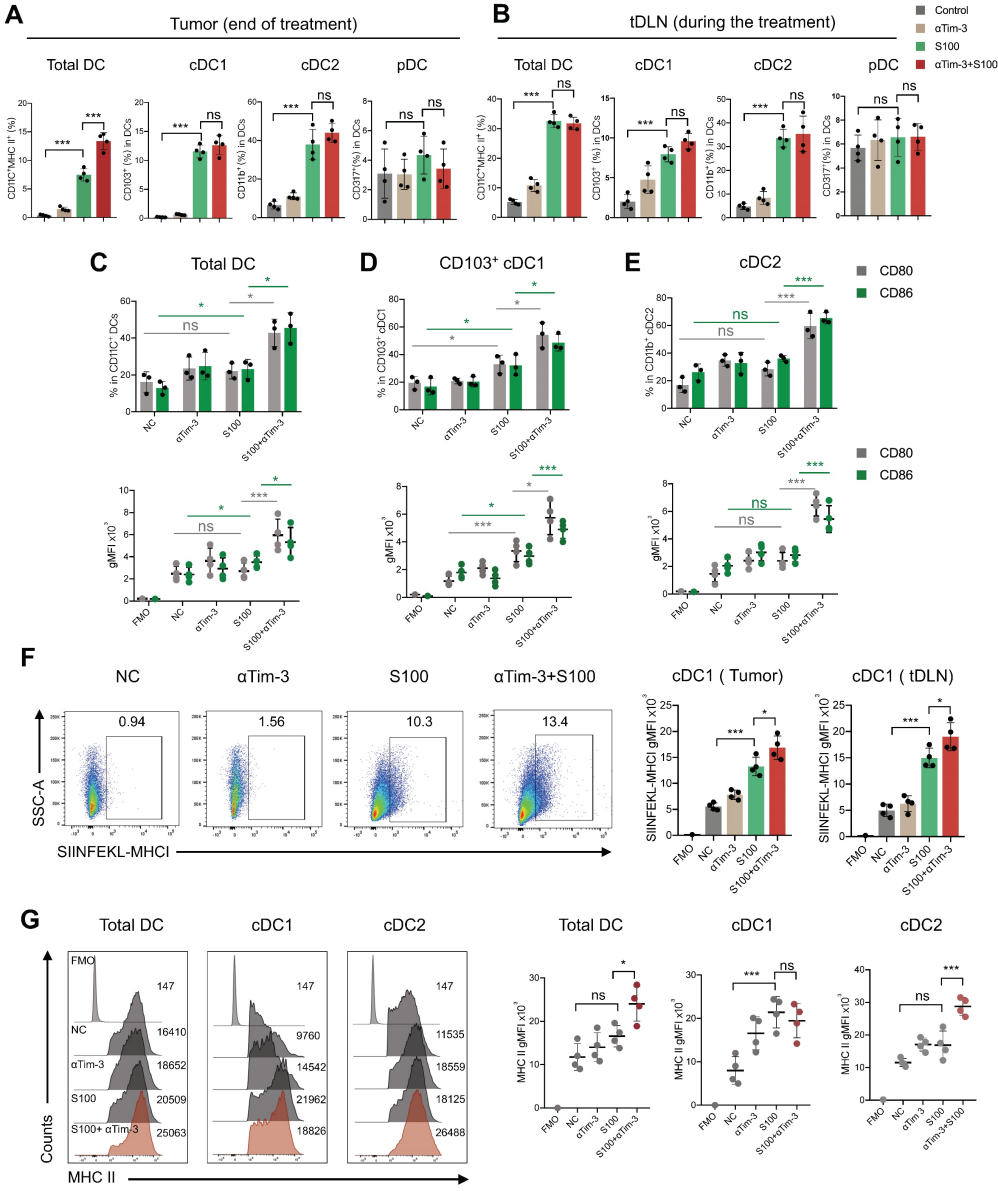
** S100 is able to activate cDC1 but fails to initiate cDC2.** Quantification of total DC, cDC1, cDC2, and pDC infiltrated in (**A**) tumors, and (**B**) tDLNs of mice bearing 4T1 from different treatment groups (n = 4 per group). Tumors and tDLNs were collected and dissected at the indicated times, while DCs were quantified by flow cytometry. Expression of classic co-stimulatory molecules CD80 and CD86 in tumor-infiltrating total (**C**) DC, (**D**) cDC1, and (**E**) cDC2 of mice bearing 4T1 from different treatment groups. Tumors were collected and DCs were analyzed as in (**A-B**). (**F**) Expression of the OVA peptide SIINFEKL-MHC-I complex on cDC1 infiltrated in tumors and tDLNs of mice bearing B16F1-OVA tumor in different treatment groups (n = 4 per group). (**G**) Expression of MHC II in tumor-infiltrating total DC, cDC1, and cDC2 of mice bearing 4T1 from different treatment groups. Tumors were collected and DCs were analyzed as in (**A-B**). S100, ADU-S100; αTim-3, anti-Tim-3; cDC, conventional dendritic cell; tDLN, tumor draining lymph node; MHC, major histocompatibility complex. Data are presented as means ± SD. *p < 0.05; ***p < 0.01; ns, not significant. Unpaired two-tailed Student's t-test.

Accordingly, we propose that the DCs must play an important role in the immunity response elicited by S100 combined with αTim-3. First, DCs infiltrated in tumors and tDLNs of mice in different treatment groups were investigated. Tumors and tDLNs were collected and dissected, DCs were quantified via flow cytometry, and the gating strategy for the characterization of cDC1 and cDC2 was provided in Figure S6. Compared with the control and αTim-3 monotherapy, both S100 monotherapy and combination therapy markedly expanded the infiltration of total DCs, as well as the subtypes cDC1 and cDC2, while plasmacytoid DC (pDC) remained stable (Figure 3A-B). Considering that pDC is known for the capacity to promote anti-viral immune responses, in addition to showing minimal impacts in monotherapy and combined therapy, pDC was excluded from being a major target of this research. The maturation and immune function of tumor-infiltrating DCs were then tested, considering that IFN-I, which is downstream of the STING pathway, could greatly induce DC infiltration and maturation 29. We hypothesized that the STING agonist S100 would make a substantial contribution to DC maturation. Surprisingly, only cDC1 from tumors isolated from the S100-treated mice displayed enhanced maturation compared to that of the control (Figure 3D). Total DCs, especially cDC2, did not elicit an up-regulation of DC classic co-stimulatory molecules CD80 and CD86, suggesting the maturation of cDC2 was not enhanced by S100 (Figure 3C-E). Importantly, with the addition of αTim-3, CD80 and CD86 showed a boost expression in total DCs and both the subtypes cDC1, cDC2 (Figure 3C-E). Noting this inconsistency of cDC1 and cDC2, the impacts of S100 on DCs were investigated further. Results showed that increased SIINFEKL-MHCI complex on cDC1 from tumors and tDLNs of mice bearing B16-OVA tumors were observed both in S100 monotherapy and combination therapy groups, indicating the enhanced peptide loading and antigen presentation capability of cDC1 (Figure 3F). Tumor antigen cross-presentation by DC cells in the context of the major histocompatibility complex (MHC) is generally considered a prerequisite for priming T cell response. Flow cytometry was conducted to explore the MHC II molecules expressed on total DC and subtypes. Total DCs, especially cDC2, did not show an up-regulation of MHC II, suggesting the peptide loading and antigen presentation capability of cDC2 was not enhanced by S100 (Figure 3G), while a significant up-regulation of MHC II was observed when αTim-3 was added (Figure 3G). Taken together, these results suggested that STING agonist S100 had the capacity to promote cDC1 maturation and activation, but displayed minimal effect on cDC2, with the addition of αTim-3, cDC2 can be initiated.

### S100 increased Tim-3 expression on cDC2 and then subdued CD4^+^ T cell response

**Figure 4:**
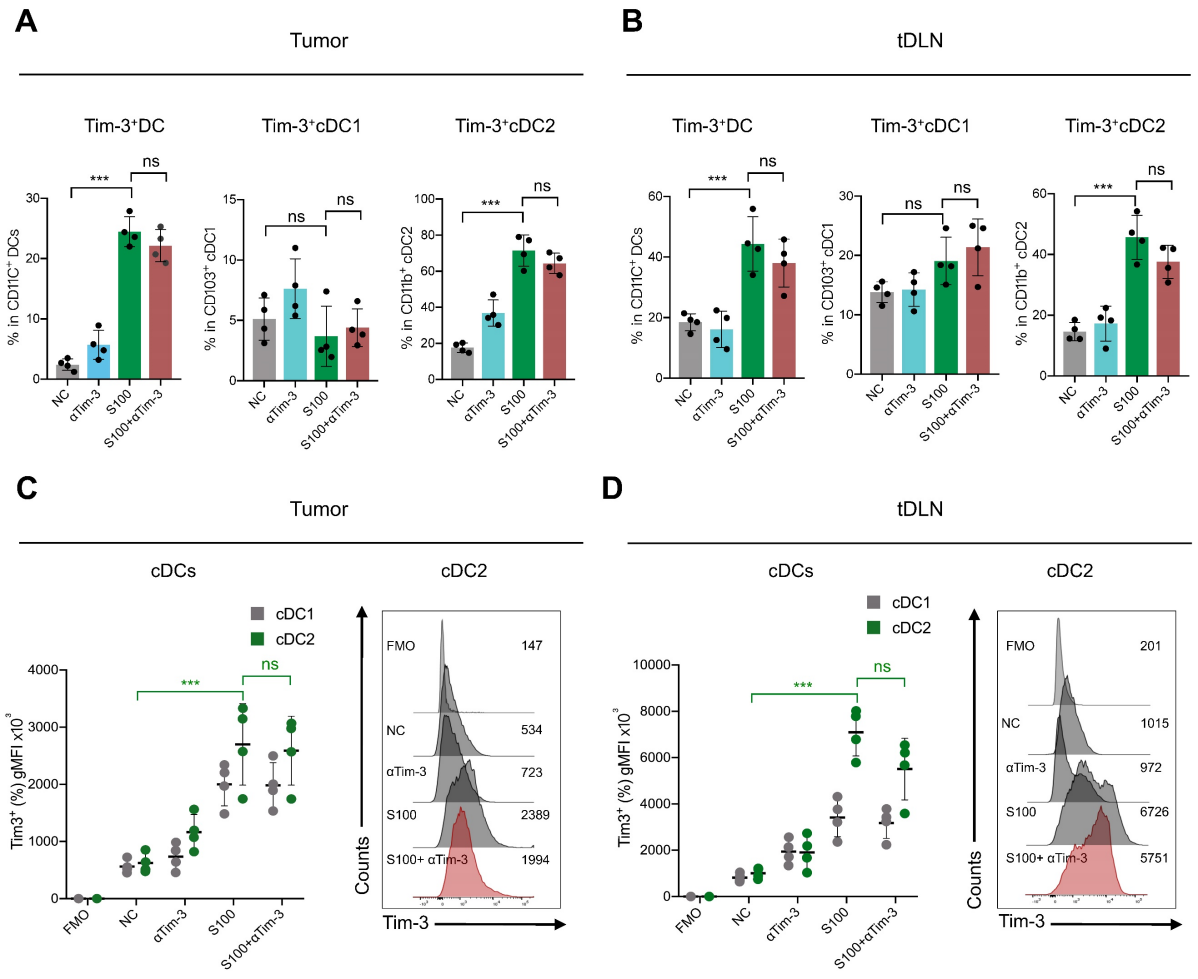
** S100 significantly increased the expression of Tim-3 on DCs, mainly cDC2.** Expression of Tim-3 on total DC, cDC1 and cDC2 infiltrated in (**A**) tumors and (**B**) tDLNs of mice bearing 4T1 from different treatment groups (n = 4 per group). Tumors and tDLNs were collected and dissected at the indicated times, while DCs were quantified using flow cytometry. **C-D**,** g**MFI of Tim-3 induced by S100 expressed on (C) tumor-infiltrating DCs and (D) DCs in tDLNs and analyzed using flow cytometry. S100, ADU-S100; αTim-3, anti-Tim-3; tDLN, tumor draining lymph node; gMFI, geometric mean fluorescence intensity. Data are presented as means ± SD. *p < 0.05; ***p < 0.01; ns, not significant. Unpaired two-tailed Student's t-test.

To further reveal the effect of S100 on cDC2, the tumors and tDLNs of mice from different treatments were collected to analyze the expression of critical inhibitory checkpoints. The data displayed a significant increase of Tim-3 expression on DCs, mainly on cDC2, while cDC1 had minimal impacts. For the S100 monotherapy and combination therapy groups, the percentage of Tim-3 positive cDC2 were much higher than that in control and αTim-3 monotherapy groups both in tumors and tDLNs (Figure 4A-B). These differences were significant when comparing the Tim-3 expression intensity (Figure 4C-D). In addition, Tim-3 expression on other immune cells was monitored, and NK cells and macrophages also showed an increase in Tim-3 while CD8^+^ T cells remained stable (Figure S7).

**Figure 5:**
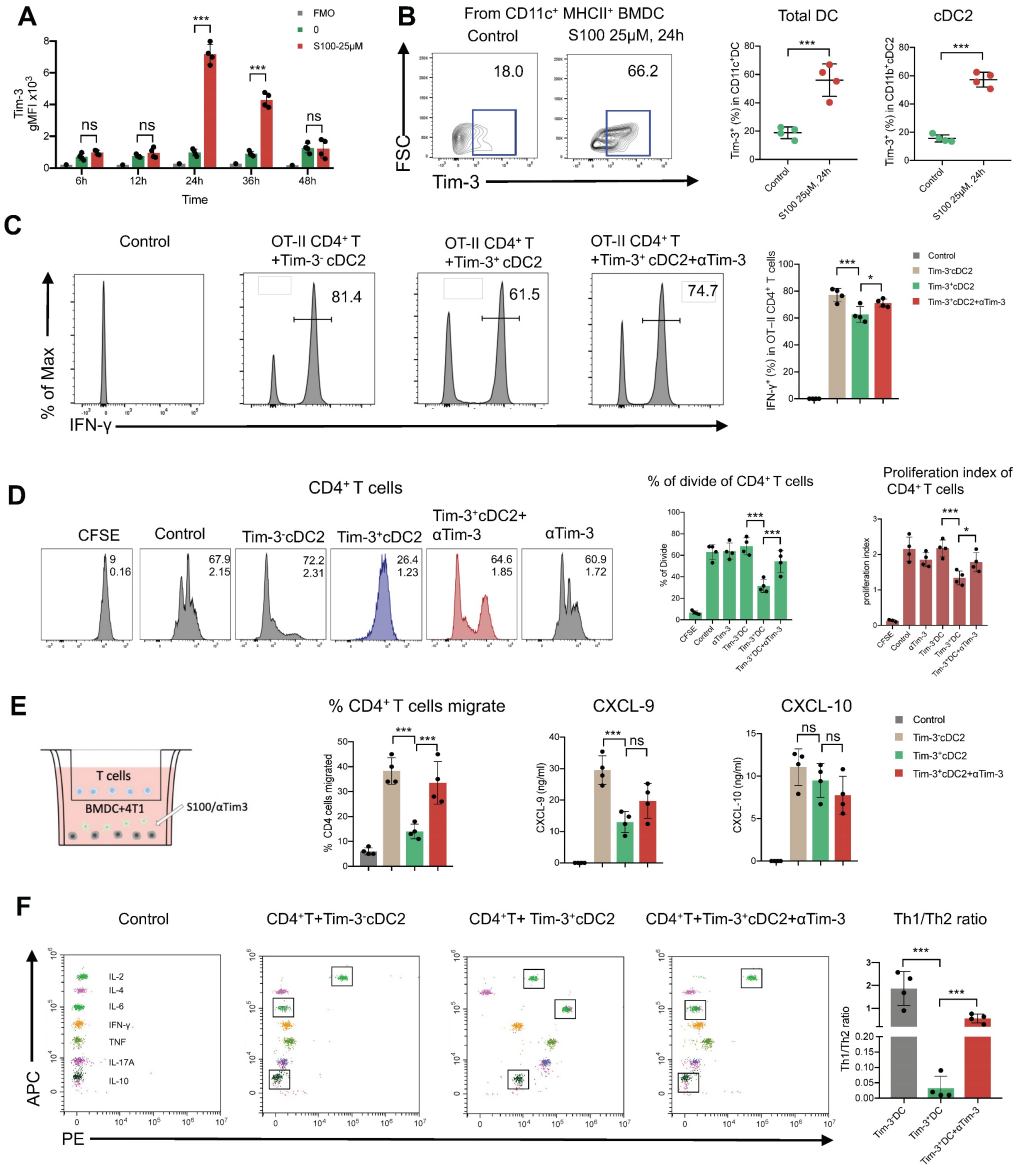
** cDC2 with high expression of Tim-3 modulated CD4^+^ T cell activity.** (**A**) S100 at 25 μM increased Tim-3 expression on DCs. DCs isolated from mature BMDCs were co-cultured with 25 μM S100 at 24 h and displayed a significant up-regulation of Tim-3. (**B**) Representative image and quantification of total DCs and cDC2 of the Tim-3 expression induced by S100. (**C**) Frequency of IFN-γ^+^ CD4^+^ T cells from OT-II CD4^+^ T cells (isolated from spleens) primed by Tim-3^-^ cDC2, Tim-3^+^ cDC2, or αTim-3 with the addition of OVA_323-339_. Frequency of IFN-γ^+^ CD4^+^ T cells was measured via flow cytometry. (**D**) Representative image and of the proliferation rate for CFSE-labeled CD4^+^ T cells incubated with Tim-3^-^ cDC2 or Tim-3^+^ cDC2 (with or without αTim-3), as well as the proliferation parameter percentage of divide and proliferation index. (**E**) Illustration of chemotaxis assay for CD4^+^ T toward Tim-3^-^ cDC2 or Tim-3^+^ cDC2 mixed tumor co-cultures (left). Quantification of CD4^+^ T cell migration in the tumor-conditioned medium in the presence of Tim-3^-^ cDC2 or Tim-3^+^ cDC2 (with or without αTim-3; middle). CXCL-9 and CXCL-10 levels in the supernatants of the co-culture system measured using ELISA (right). (**F**) Th1/Th2/Th17 cytokine profiles (left), and Th1/Th2 ratio (right). CD4^+^ T cells were incubated with Tim-3^-^ cDC2, Tim-3^+^ cDC2 (with or without αTim-3), and Th1/Th2/Th17 cytokines secreted in the supernatant and measured via flow cytometry. S100, ADU-S100; αTim-3, anti-Tim-3; cDC, conventional dendritic cell; BMDCs, bone marrow-derived DCs; CXCL-9, C-X-C motif chemokine ligand 9; Th, T helper; gMFI, geometric mean fluorescence intensity. Data are presented as means ± SD. *p < 0.05; ***p < 0.01; ns, not significant. Unpaired two-tailed Student's t-test.

Considering that Tim-3 expressed on DCs (and no other immune cells) represents a crucial negative regulation molecule, we hypothesized that Tim-3^+^cDC2, which were induced by STING agonist S100 monotherapy, represented a population of negative regulatory cells. To test this, both *in vitro* and *in vivo* investigations were conducted. First, to confirm that S100 has the ability to up-regulate Tim-3 expression on DCs, both mouse DCs and human DCs were used. BMDCs were extracted from naive mice and human DCs were generated by HSCs. Cytokines induced mature BMDCs and mature human DCs were co-cultured with different concentrations of S100, both mouse cDC2 and human cDC2 displayed a significant up-regulation of Tim-3 at 12.5-200 μM of S100 (Figure 5A-B, Figure S8, S9) lasting approximately 24 h, confirming the above results derived from murine models. Accordingly, 25 μM of S100 was chosen as the treatment concentration for subsequent functional studies in murine models. We showed that S100 has minimal effect on the cDC2 maturation and antigen presentation *in vivo*. To explore whether this was caused by the up-regulation of Tim-3 on cDC2, an *in vitro* T-cell priming model involving the activation of OT-II CD4^+^ T cells co-incubated with Tim-3^+^cDC2 (cDC2 isolated from mature BMDCs and induced by S100 for 24 h) or Tim-3^-^cDC2 pulsed with a specific peptide—ovalbumin peptide (OVA_323-339_)—was employed. As predicted, Tim-3^+^cDC2 exhibited an impaired ability to initiate the production of the autoimmune factor IFN-γ in OT-II CD4^+^ T cells, indicating the impaired T-cell priming function of DCs when Tim-3^+^cDC2 was in the co-culture system. At the same time, αTim-3-treated CD4^+^ T cells displayed a recovery of killing ability, suggesting the high expression of Tim-3 on cDC2 had a negative regulation on DC peptide loading and antigen presentation capabilities (Figure 5C).

As a population of professional antigen-presenting cells, cDC2 are required for CD4^+^ T cell priming. We confirmed that the expression of Tim-3 on cDC2 produced an inhibitory effect. To investigate the impact of Tim-3^+^ cDC2 on the proliferation of T cells *in vitro*, CD4^+^/CD8^+^ T cells (CFSE-labeled) were co-incubated with Tim-3^+^cDC2 or Tim-3^-^cDC2. CD4^+^ T cells co-cultured with Tim-3^-^cDC2 displayed a normal proliferation ability, but a delayed CFSE-labeled proliferation was observed in those co-cultured with Tim-3^+^cDC2 (Figure 5D). Meanwhile, the proliferation of CD4^+^ T cells co-cultured with Tim-3^+^cDC2 recovered with the addition of αTim-3. The proliferation of CD8^+^ T cells showed minimal impacts after the co-incubation of Tim-3^+^cDC2 (Figure S10A,); thus, we concluded that Tim-3^+^cDC2 had an inhibitory effect on the proliferation of CD4^+^ T cells.

We then questioned whether Tim-3^+^cDC2 would impact the migration ability of T cells. To assess the cell migratory potential, *ex vivo* transwell assays were performed. We observed that CD4^+^ T cells migrated significantly when Tim-3^-^cDC2 were co-cultured with tumor cells, whereas Tim-3^+^cDC2 co-cultured with tumor cells displayed reduced chemotaxis on CD4^+^ T. Following the addition of αTim-3, CD4^+^ T cells showed an enhanced migration ability compared to those cultured with Tim-3^+^cDC2 (Figure 5E). Similarly to the findings from CFSE-labeled proliferation, the migration ability of CD8^+^ T was only slightly affected (Figure S10B); thus, the transwell assays indicated that the migration ability of CD4^+^ T cells (but not CD8^+^ T cells) was hindered by Tim-3^+^cDC2.

The above results begged the question whether chemokines were differently secreted by DCs with Tim-3^+^ or Tim-3^-^. Accordingly, ELISA was performed to analyze the most common T cell chemokines, CXCL9 and CXCL-10. The expression of CXCL-9 was significantly decreased from Tim-3^+^cDC2 (Figure 5E); thus, CXCL-9 secretion was decreased in cDC2 with Tim-3^+^, hindering the migration of CD4^+^ T cells.

CD4^+^ Th cells were thought to be limited to two major subsets—Th1 and Th2—based on their production of specific cytokines (IFN-γ/IL-2 and IL-6/IL-10, respectively). The Th1/Th2 paradigm is another critical factor effecting CD4^+^ Th cell immune functions. To this end, we investigated whether Tim-3^+^cDC2 would influence the Th1/Th2 balance of CD4^+^ Th cells. In the co-culture system, including tumor cell (4T1), CD4^+^ T cells, and Tim-3^+^ cDC2/Tim-3^-^ cDC2, the cytokines secreted in the supernatant were analyzed; revealing that the levels of IL-6 and IL-10 were substantially higher in the Tim-3^+^cDC2 co-culture system and indicating an induced increase of Th2 cells. Similarly, a reduction of IL-6 and IL-10 levels was observed with the addition of αTim-3 (Figure 5F). These results showed that Tim-3^+^cDC2 had the potential to induce CD4^+^ Th cells to Th2 cells, considered a negative effect on CD4^+^ T cells. In conclusion, S100 up-regulated the expression of Tim-3 on cDC2, representing a novel negative regulatory effect on CD4^+^ T cells. In addition, RNA-seq was performed further address the singular differences in gene expression between Tim-3^+^ cDC2 and Tim-3^-^ cDC2 through GO analysis. In the dataset, we identified 200 DEGs in Tim-3^+^cDC2 compared to Tim-3^-^cDC2 (111 upregulated genes and 89 downregulated genes). Details on the DEGs are provided in the Supplementary material (Table S1). Several immune-related genes, such as TREM2, Ffar2, HAVCR2, and HAVCR1 were up- or downregulated (Figure S11A). GO function analysis identified nine BPs, five MFs, and four CCs that were markedly enriched. In Tim-3^+^cDC2, the top-ten ranked BP terms included calcium-mediated signaling, response to interleukin-6, neutrophil chemotaxis, myeloid dendritic cell activation, and leukocyte differentiation. Moreover, significantly enriched CC and MF terms were found (Figure S11B). Overall, the BP, MF, and CC terms that were significantly enriched in Tim-3^+^ cDC2 were relatively different from those in Tim-3^-^ cDC2.

### αTim-3 significantly promoted anti-tumor immunity of S100 by unleashing CD4^+^ T cells

**Figure 6:**
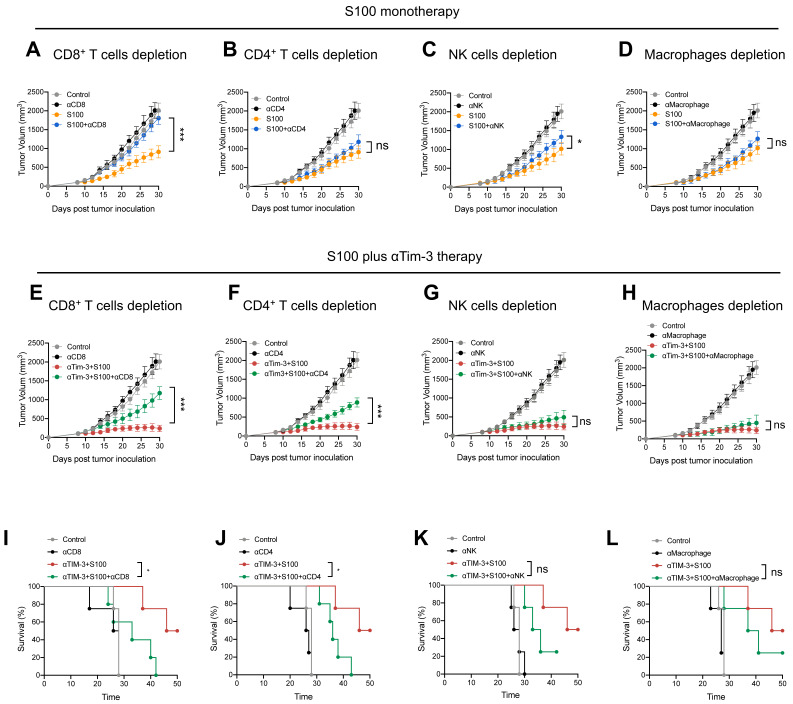
** αTim-3 significantly promoted the anti-tumor response of S100 by unleashing CD4^+^ T cells.** Tumor volume of mice bearing 4T1 tumor treated with S100 monotherapy, either with or without depletion antibodies against (**A**) CD8^+^ T cells, (**B**) CD4^+^ T cells, (**C**) NK cells, (**D**) or macrophages (n = 4 mice per group). Only CD8^+^ T cells are important for S100 monotherapy in tumor treatment. Tumor volume of mice bearing 4T1 tumor treated with combination treatment S100 plus αTim-3, either with or without depletion antibodies against (**E**) CD8^+^ T cells, (**F**) CD4^+^ T cells, (**G**) NK cells, or (**H**) or macrophages (n = 4 mice per group). Both CD8^+^ T cells and CD4^+^ T cells are important for the combination treatment. Survival of mice bearing 4T1 tumors treated with the combination treatment S100 plus αTim-3, either with or without depletion antibodies against (**I**) CD8^+^ T cells, (**J**) CD4^+^ T cells, (**K**) NK cells, or (**L**) macrophages (n = 4 mice per group). S100, ADU-S100; αTim-3, anti-Tim-3; NK, natural killer. Data are presented as means ± SD. *p < 0.05; ***p < 0.01; ns, not significant according to an unpaired two-tailed Student's t-test.

The negative effect of Tim-3^+^cDC2 on CD4^+^ T cells was also evaluated, revealing that αTim-3 could reverse these detrimental impacts, and unleash CD4^+^ T cells. To confirm whether the CD4^+^ T cells were the primary mechanism by which αTim-3 enhanced the anti-tumor immunity of S100, antibody blockade assays were performed, revealing the indispensable role of adaptive immunity for CD4^+^ T cells in the combination of αTim-3 with S100. First, to evaluate the immune cell-dependence of S100, 4T1 tumor growth inhibition was evaluated via the antibody blockade of CD4^+^ T cells (anti-CD4 antibody, αCD4), CD8^+^ T cells (anti-CD8α antibody, αCD8), NK cells (anti-Asialo-GM1 antibody, αNK), and macrophages (clodronate liposomes, αMacrophage) in the STING agonist S100 monotherapy group (Figure 6A-D). Immune cell depletion efficacy is displayed in Figure S12. The results obtained from mice treated with antibodies in the S100 monotherapy group showed that blocking CD8^+^ T cells resulted in the significant inhibition of tumor growth at late time points (Figure 6A); whereas the blockage of CD4^+^ T cells (Figure 6B), NK cells (Figure 6C), and macrophages (Figure 6D) produced only minimal effects, suggesting that S100 primarily exerted an anti-tumor immunity via CD8^+^ T cells. By contrast, the results of antibody blockade assays in the combination treatment group were distinct. Blocking of either CD8^+^ T (Figure 6E) or CD4^+^ T cells (Figure 6F) resulted in the partial inhibition of tumor growth, while the depletion of NK cells (Figure 6G) or macrophages (Figure 6H) did not affect tumor growth inhibition, indicating that CD8^+^ and CD4^+^ T both played important roles in anti-tumor effects. Compared with S100 monotherapy, CD4^+^ T cells showed little effect on the anti-tumor immunity of S100; whereas with the addition of αTim-3, CD4^+^ T cells contributed to an anti-tumor response. This suggests that CD4^+^ T are primarily responsible for the αTim-3-enhanced anti-tumor response of S100. Furthermore, like mice treated with αCD8, those treated with αCD4 (Figure 6I-J) in the combination group had a worse survival rate than those treated with αNK or αMacrophage (Figure 6K-L), demonstrating that CD4^+^ T played an important role in the S100/αTim-3 combination treatment. To further address the singular importance of Tim-3 on dendritic DCs rather than other immune cells, we performed an *in vivo* adoptive transfer of immune cells with Tim-3 high expression and an *in vitro* CD4^+^ T cell priming assay. BMDCs, BMDMs, NK cells and CD8^+^ T cells were co-cultured with S100, and then Tim-3 expression was detected using flow cytometry. Immune cells with high or low Tim-3 expression were intravenously injected into 4T1 tumor-bearing mice, the results showed that the injection of Tim-3^+^cDC2 exhibited an impaired ability to initiate the production of IFN-γ in CD4^+^ T cells compared with the injection of Tim-3^-^cDC2. In contrast, the injection of other immune cells (CD8^+^ T, BMDM, and NK) with high or low Tim-3 expression did not affect the production of IFN-γ in CD4^+^ T cells (Figure S13). To further confirm that other immune cells with high or low Tim-3 expression did not influence the cytotoxicity of CD4^+^ T cells, an *in vitro* T-cell priming model was employed involving CD4^+^ T cells that were co-incubated with CD8^+^ T, BMDM, and NK (with high or low Tim-3 expression) and co-cultured with tumor cells for 24 h. As predicted, the expression of Tim-3 on these immune cells did not affect the production of IFN-γ in CD4^+^ T cells, indicating that Tim-3 expressed in CD8^+^ T, BMDM, and NK immune cells did not impair the CD4^+^ T-cell priming functions compared with the findings shown in Figure 5C of the manuscript. Thus, Tim-3 expressed on DCs (but not on T cells, NK cells, or macrophages) negatively regulated CD4^+^ T cells (Figure S14).

### High percentages of Tim-3^+^cDC2 predicted poor prognosis in patients

**Figure 7:**
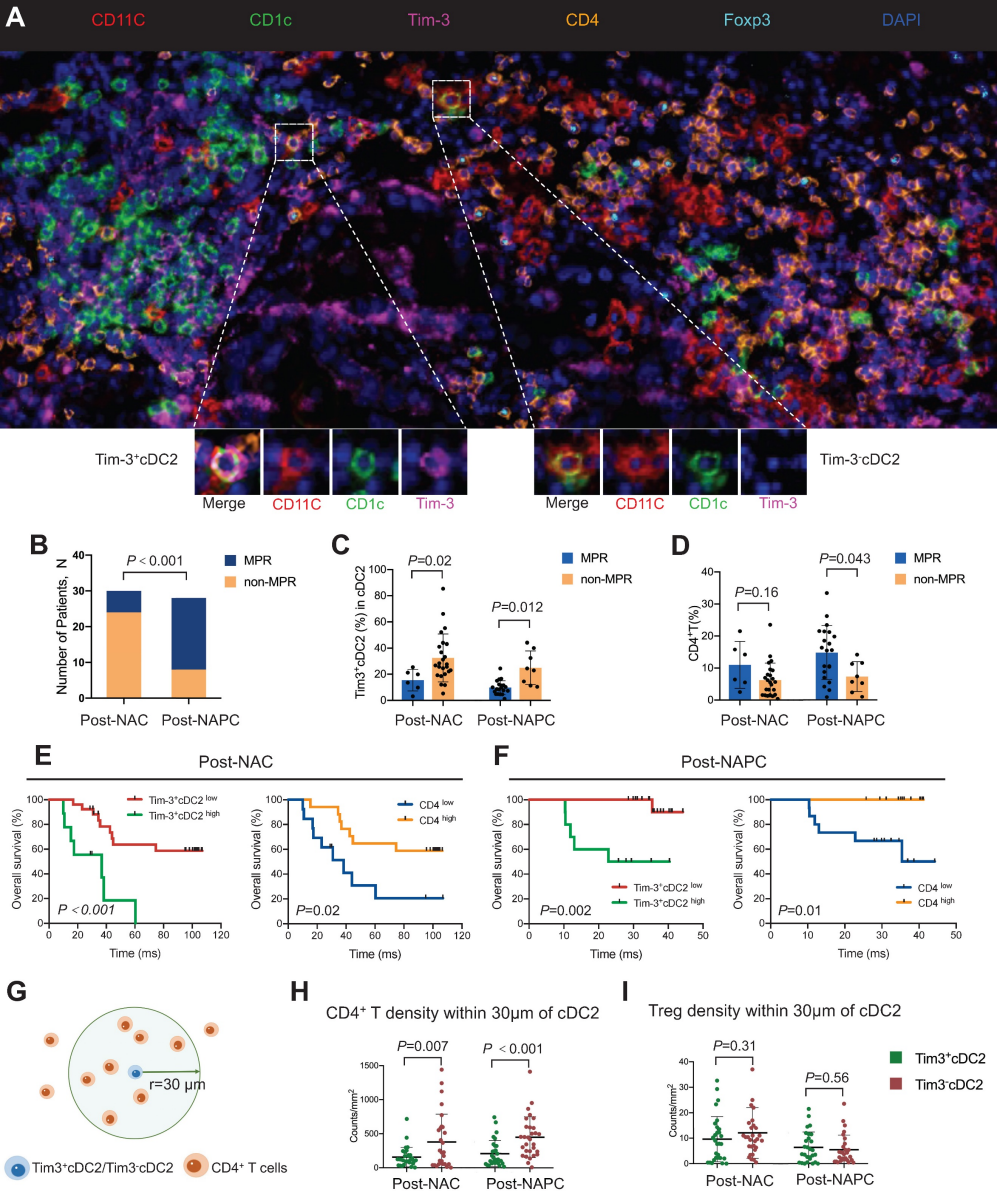
** High percentages of Tim-3^+^cDC2 predicted poor prognosis in tumor patients.** (**A**) Representative mIHC staining image (20×) of post-treatment tumor samples. (**B**) Comparison of MPR percentage for patients treated with NAC or NAPC. Comparison of (**C**) Tim-3^+^cDC2, or (**D**) CD4^+^ T for patients who displayed MPR or non-MPR treated with NAC or NAPC. Comparison of OS for patients with different Tim-3^+^cDC2 or CD4^+^ T statuses treated with (**E**) NAC or (**F**) NAPC. (**G**) Illustration of methodology for spatial analyses performed. Densities of interest cells within a certain radius (30 μm) to a reference cell were calculated. Density of (**H**) CD4^+^ T and (**I**) Treg cells within 30 μm of Tim-3^-^cDC2 cells, compared with Tim-3^+^cDC2 cells in NAC and NAPC patients. S100, ADU-S100; αTim-3, anti-Tim-3; cDC, conventional dendritic cell; Treg, regulatory T cells; mIHC, multiplex immunohistochemistry; MPR, major pathological response; NAC, neoadjuvant chemotherapy; NAPC, neoadjuvant pembrolizumab and chemotherapy; OS, overall survival. Log-rank tests and unpaired two-tailed Student's t-tests were used to determine statistical significance. Data are presented as means ± SD. *p < 0.05; ***p < 0.01.

Based on the aforementioned results, tumor samples were collected from 58 patients with lung cancer who received NAC or NAPC. mIHC was conducted, and the relationships between the tumor-infiltrating Tim-3^+^ cDC2 or CD4^+^ T cells and the therapeutic response were evaluated. Representative images are provided in Figure 7A and Figure S15. The clinicopathological characteristics of the included patients are described in Table 1. Patient characteristics were generally balanced between the two groups, where those treated with NAPC obtained a higher major pathological response (MPR) than those treated with NAC, indicating that NAPC improved pathological response in patients with NSCLC (Figure 7B). The results showed that patients who did not achieve MPR had a higher percentage of Tim-3^+^ cDC2 in both the NAC and NAPC groups, with these differences being statistically significant (p = 0.02 and 0.012, respectively). Thus, Tim-3^+^cDC2 appeared to predict a worse therapeutic response (Figure 7C). The results of the CD4^+^ T population differed completely, as patients who did not achieve MPR appeared to have a lower tumor-infiltration of CD4^+^ T cells (p = 0.043 in NAPC patients), although differences for NAC patients were not statistically significant (p = 0.16 in NAPC, Figure 7D). In addition to the therapeutic response, poor survival also appeared to be related to Tim-3^+^cDC2. To this end, patient follow-ups were conducted from January 1, 2014 to October 10, 2022, with a median follow-up time of 97 months for post-NAC patients, and 35 months for post-NAPC patients. The overall survival (OS) and progression-free survival (PFS) were observed and analyzed. The median percentage of Tim-3^+^cDC2 was used as the cutoff value of total cDC2, where patients with a higher (or equal) percentage of Tim-3^+^cDC2 than the median were considered as Tim-3^+^cDC2^high^, and the remainder were characterized as Tim-3^+^cDC2^low^. Survival analyses revealed that compared with Tim-3^+^cDC2^low^ patients, Tim-3^+^cDC2^high^ patients had a shorter OS, with these differences being statistically significant (p < 0.001 for NAC patients, and p = 0.002 for NAPC patients, Figure 7E-F). The relationship between the tumor-infiltrating CD4^+^ T cells and survival was also assessed. Here, inverse data were found compared with Tim-3^+^cDC2, as CD4^high^ patients had a better OS (p = 0.02 for NAC patients, and p = 0.01 for NAPC patients; Figure 7E-F). Furthermore, patients who had lower Tim-3^+^cDC2 and higher CD4^+^ T cells appeared to have improved PFS in the NAPC group, whereas the differences for NAC patients were not significant (Figure S16).

Considering the above results, and that CD4^+^ T cells are down-stream of cDC2, there is likely an interaction between CD4^+^ T cells and cDC2. To quantify this interaction, the spatial distribution between Tim-3^-^cDC2/Tim-3^+^cDC2 and CD4^+^ T/Treg cells was explored. The bivariate K(r) function 30 was used to characterize the spatial distributions for each two phenotypes of cDC2 and CD4^+^ T cells. The radius used (r = 30 μm) is generally considered ideal for calculating the spatial relationship between two cell populations (Figure 7G-I). A significant reduction in CD4^+^ T cells around Tim-3^+^cDC2 compared with Tim-3^-^cDC2 was observed in both NAC and NAPC patients, with these differences showing statistical significance (p = 0.007 and < 0.001, respectively), representing a decreased probability of cell-cell contact between CD4^+^ T and Tim-3^+^cDC2. As a type of classical immune-suppressive cell, Treg did not exhibit a clear spatial relationship with Tim-3^+^cDC2. In conclusion, this tumor sample analysis provided preliminary evidence supporting the relationship of Tim-3^+^cDC2 with poor prognoses in tumor patients, and relevant clinical trials are urgently required.

It was reported that CD4^+^ T cell activation was initiated when a TCR recognized a specific peptide presented on MHC-II expressed by DCs. Following DC antigen presentation, TCR had the potential to make conformational changes 31. Based on single-cell sequencing data, TCR indices including Shannon index were then calculated in both Tim-3^+^cDC2^high^ and Tim-3^+^cDC2^low^ patients to determine TCR diversity. We found that these indices were highly sensitive to low-frequency clones, while clonality (which was closely related to expanded clones) and richness reflected the actual number of unique TCR sequences 32. The expression of Tim-3 on cDC2 had a negative influence on TCR clonality, as patients with higher Tim-3^+^cDC2 maintained decreased clonality. In analyzing the correlation between TCR clonality and Tim-3^+^cDC2 in patients, we found that the two showed a weak negative correlation (Figure S17; r = -0.65), although this relationship was not significant, likely owing to the limited sample number. Furthermore, it was found that TCR diversity or richness were not associated with patient percentage of Tim-3^+^cDC2. Taken together, these data indicated that the high expression of Tim-3 on cDC2 may inhibit the CD4^+^ T cell clonal expansion, consistent with prior results obtained from the murine models where it was revealed that Tim-3^+^cDC2 could impair the proliferation of CD4^+^ T cell.

## Discussion

Recently, the application of immune checkpoint inhibitors (ICIs), such as cytotoxic T lymphocyte-associated protein 4 (CTLA-4) or PD-1, have produced significant improvements in anti-tumor treatment 33; however, refractory or relapse after CPI immunotherapy is frequent in patients, necessitating the investigation for new strategies to heighten anti-tumor responses 34. The cGAS-STING pathway is a type of DNA sensor, required for the spontaneous generation of tumor-specific immune responses in the tumor setting 35. Based on the critical role of the STING axis for the induction of anti-tumor immunity, STING agonists are presently being explored as an anti-tumor therapy strategy 36. Specifically, the STING agonist S100, also called MIW815 was developed with rational and effective chemical modifications of natural STING ligands to enhance stability and facilitate the activation of all five common human STING alleles 37, 38. In S100 monotherapy, intratumoral (IT) injection of S100 presented potent anti-tumor immunity in various murine tumor models 39-41. In light of promising preclinical data, S100 has been long considered a promising anti-tumor agent 42. As the first candidate to move to early clinical investigation (NCT: NCT02675439 and NCT03172936) 8, S100 unexpectedly failed to show satisfactory anti-tumor effects in human patients, for both single agent and when combined with anti-PD-1; thus, the investigation of S100 has moved at a relatively slow pace. Herein, it is reported that the block of Tim-3 enhances the anti-tumor immunity of STING agonist ADU-S100 by unleashing CD4^+^ T through targeting Tim3^+^cDC2. First, the combination of αTim-3 with S100 was investigated, and revealed that tumor growth *in vivo* was significantly delayed, and toxicities were acceptable. Next, we observed that the antigen-specific T cells were apparently increased and activated in the combination therapy group; leading to the question of whether the DCs played an important role in the combination of S100 plus αTim-3. In the S100 monotherapy group, the maturation and antigen presentation of cDC2 were not initiated, while cDC1 were boosted, but in the combination group, the addition of αTim-3 significantly promoted cDC2 maturation and antigen presentation. These results strongly suggested that S100 monotherapy may partly suppress the cDC2 function, whereas blocking Tim-3 could counteract this suppression. Further investigations revealed that S100 could upregulate the expression of Tim-3 on cDC2. Tim-3 was reported to have specific significance on DCs, promoting immune evasion and blocking Tim-3 on DC to facilitate anti-tumor immunity. Based on this and the present flow cytometry analyses showing Tim-3 was highly expressed on cDC2, we hypothesized that Tim-3^+^cDC2 formed a population of suppressive immune cells. To further understand the function of Tim-3^+^cDC2, Tim-3^+^cDC2 induced by S100 were prepared, and *in vitro* experiments were performed. The findings indicated that the high expression of Tim-3 negatively regulated cDC2 and CD4^+^ T downstream. This was in agreement with previous findings that block the Tim-3 on DC-cytokine induced killer (CIK) cells with antibodies, which can enhance the killing ability of DC-CIK cells 43. Consistently with the findings of our present study, S100 has been reported to exhaust CD4^+^ T cells in mice 44. Thus, the results presented here showed that Tim-3^+^cDC2 and its downstream CD4^+^ T cells may be the key immune factors for combination therapy. A subsequent *in vivo* immune cell depletion experiment similarly confirmed the significant role of CD4^+^ T in combination. As a whole, the data indicated that the activation of CD4^+^ T, rather than CD8^+^ T was the potential reason why blockage of Tim-3 could significantly enhance the anti-tumor immunity of S100. Notably, CD8^+^ T subsets also make a contribution to the anti-tumor immunity both in S100 monotherapy or the combination therapy; however, they are not the major target rescued by αTim-3.

During these years, CD8^+^ T cells were regarded as a primary immunotherapeutic target based on their classic role in cytotoxicity towards tumor cells 45. Presently, CD4^+^ T cells are rapidly emerging as another important contributor to anti-tumor response 46, 47. In the TME, CD4^+^ T cells have been shown to enhance immunity and stimulate pro-inflammatory myeloid cell programs 48. It has also been reported that CD4^+^ subsets improve the quality of CD8^+^ subset response to tumor antigens, and contribute to T cell memory programming and maintenance 49, 50. Recently, in the ICIs treatment of cancer, CD4^+^ T cells also played an important role as a predictive index of therapeutic outcomes 48. Our previous study found that CD4^+^ T cells are essential for CIK treatment, capable of reversing functional exhaustion and restoring the cytotoxicity of CIK cells 51. Fan et al. 52 reported that α-CTLA-4 therapy led to a systemically circulating population of specific CD4^+^ Th1-like effector CD4^+^ T, components that are critical for antitumor response. Conversely, CD4^+^ T, with high expression of PD-1, were considered exhausted and were found to be a negative prognostic indicator for ICI therapy 53; thus, the biological activities that contribute to the activation of anti-tumor CD4^+^ T merit further investigation. Here, we found that S100 can significantly promote CD8^+^ T, but restrict CD4^+^ T, the latter of which may be the key factor controlling the unsatisfactory results of S100 to date. Experiments performed *in vivo* using immune cell depletion antibodies or agents showed that in the S100 monotherapy group, blockage of CD8^+^ T cells resulted in the significant inhibition of tumor growth; whereas CD4^+^ T cells, macrophages, and NK cells elicited minimal effects, suggesting that S100 exerted an anti-tumor immunity which was primarily dependent upon CD8^+^ T cells. Interestingly, the results of antibody blockade assays in the combination treatment group were different, where blocking either CD8^+^ T or CD4^+^ T cells resulted in the partial inhibition of tumor growth, indicating the CD8^+^ and CD4^+^ subsets each played important roles in anti-tumor effects. Compared with S100 monotherapy, CD4^+^ T cells showed little effect on the anti-tumor immunity of S100; whereas with the addition of αTim-3, CD4^+^ T cells contributed to the anti-tumor response. It has previously been reported that CD4^+^ T cells can positively regulate the function of CTL by enhancing their activity, migration, and survival in tumors 47, 49. CTL maintained a stronger activation in the combination treatment here, potentially a result of unleashing CD4^+^ T. Comparing the combination with monotherapy, we discovered that the former boosted CD4^+^ T cells, while S100 induced high expression of Tim-3 on cDC2 restraining cDC2 and its downstream CD4^+^ T. To unleash CD4^+^ T cells during the S100 treatment, DCs are critical targets, and cDCs, a major subset of DCs, have the most potent antigen presentation abilities 54. In addition, during antigen-specific T cell immune responses, they cross-present tumor antigens, co-stimulatory molecules, and cytokines to trigger antigen-specific T cell initiation 55, 56. Accordingly, improvement in cDC quantity and functionality has long been considered to be therapeutically important for enhancing the effector potential of T cells 57, 58. Treatment strategies, including DC vaccines, still maintain an uncertain therapeutic effect 59, while approaches which can enhance DCs are urgently needed. cDCs can be broadly divided into two types: cDC1 and cDC2. cDC1 and cDC2 often take on specialized roles in CD8^+^ T and CD4^+^ T cell priming processes through MHC I or MHC II antigen-presenting cells 47, 60. cDC1 were the most widely studied DC subset, whose major efforts have been investigated for their critical role in defense against cancer 61. Critically, in terms of antigen presentation, cDC2 contain substantial heterogeneity 47, and have received limited attention. Based on the high capacity to cross-present antigens through MHC II, cDC2 excelled at activating CD4^+^ T cells, yet the specific roles of cDCs in eliciting antitumor CD4^+^ T immunity remains unclear 62. Here, we highlight cDC2 with high expression of Tim-3 as a means of suppression, and an important population for directing antitumor CD4^+^ T immunity.

Furthermore, in the present research, the abundance of Tim-3^+^cDC2 in human TME may act as a biomarker for CD4^+^ T quality, as well as a contributing indicator for responsiveness to immunotherapy. The clinical data presented here strongly suggested that cancer patients who did not achieve MPR had a greater percentage of Tim-3^+^cDC2 (p < 0.02 for NAC patients, and < 0.012 for NAPC patients), lower OS (p < 0.001 for NAC patients, and p = 0.002 for NAPC patients), and PFS. In DC subsets, cDC1 were associated with improved overall survival in cancer patients 63, and it has been shown that the expression of cDC2 gene signatures correlated with positive prognoses 64, while cDC2 with high expression of Tim-3, however, predicted poor survival, indicating Tim-3^+^cDC2 is an immune-suppressive cell population. Most results obtained to date have supported the hypothesis that the expression of Tim-3 on DCs predicts negative immune response 18, 43; yet, the findings here represent the first time this has been shown to be related to therapeutic outcomes and survival. Therapeutic strategies of ICIs are clinically complex, while biomarkers capable of identifying and predicting treatment responses are lacking. The possible mechanism underlying the role of Tim-3^+^cDC2 in therapeutic response pertains to the further suppression of CD4 ^+^ T cell-mediated immunity. It has previously been reported that the density of cDC2 alone correlates with abundant CD4^+^ T cells 47 which agrees well with the findings from the present study. Notably, there was a significantly downregulation in the distribution of CD4^+^ T around Tim-3^+^cDC2 compared with Tim-3^-^cDC2, indicating Tim-3^+^cDC2 negatively regulated CD4^+^ T.

The present study had several limitations. It was found that STING agonist ADU-S100 could upregulate Tim-3 on cDC2 and subdued CD4^+^ T cell response. To some extent, this may contribute to the clinical resistance of S100 systemic treatment. A single STING agonist was used in the present research, while other effective molecules, such as cGAMP or MSA-2 were not involved. Accordingly, further investigations are needed to include other STING agonists, as well as chemotherapy drugs. Moreover, in the *in vivo* experiments, cDC2 gene knockout (Irf4^-/-^) mice were not used due to the restrictions of Tim-3 expression. Comparatively, when cDC2 knockout was used, both Tim-3^+^cDC2 and Tim-3^-^cDC2 were deleted, leading to a complete loss of normal cDC2 function, possibly having an unexpected effect on other immune cells, and confounding the present observations. It has been reported that IRF4-expressing DCs regulated CD8^+^ memory precursor cells 65, while IRF4 expressed on intratumoral Tregs displayed superior suppressive activity 66. Based on this evidence, cDC2 genes knockout (Irf4^-/-^) mice were not employed in this study.

In summary, the findings here revealed that resistance to STING agonist ADU-S100 treatment, including mono- or combination therapy with αPD-1, was attributable partly to the Tim-3^+^cDC2 induced by S100 that impeded CD4^+^ T cells. With the combination of αTim-3 with S100, CD4^+^ T were unleashed, and anti-tumor immunity was significantly enhanced. While other analyses have shown that the STING-triggered IFN response may be critical for DC function, the present study confirmed that cDC2, with high expression of Tim-3, is a cell population with immune-suppressive effect on CD4^+^ T cells. Notably, Tim-3^+^cDC2 showed that minimal impacts on CD8^+^ T cells do not mean that CD8^+^ T is unimportant. Rather, CD8^+^ T subsets are critical to the anti-tumor immunity of S100; however, they are not the major target rescued by αTim-3. This study presents a previously underappreciated combination scenario, revealing an intrinsic barrier to STING agonist ADU-S100 anti-tumor immunity, and providing a combinatorial strategy to overcome the immunosuppression in tumors.

## Supplementary Material

Supplementary figures and tables.

## Figures and Tables

**Table 1:** Clinicopathological characteristics of NSCLC patients treated with NAC or NAPC.

Characters		NAC (N= 30)	NAPC (N= 28)	*p* value
Age, y				0.17
	≤60	13	7	
	>60	17	21	
Gender				0.9
	Male	26	24	
	Female	4	4	
Smoking index				1.0
	≤400	15	14	
	>400	15	14	
Pathology				0.69
	Squamous	17	19	
	Adenocarcinoma	11	7	
	Large cell	2	1	
	Sarcomatoid	0	1	
Stage				0.6
	I	1	0	
	II	6	5	
	III	23	23	
Neoadjuvant regimen				N/A
	PTX+CBP	20	N/A	
	PEM+CBP	9	N/A	
	PTX+CBP+Pembro	N/A	21	
	PEM+CBP+Pembro	N/A	6	
	Other	1	1	
Type of resection				0.43
	Pneumonectomy	7	3	
	Lobectomy	20	21	
	sleeve lobectomy	3	4	
Adjuvant therapy				0.28
	Yes	9	5	
	No	21	23	

NSCLC, non-small cell lung cancer; NAC, neoadjuvant chemotherapy; NAPC, neoadjuvant pembrolizumab and chemotherapy; y, year.

## Abbreviations

TME: immunosuppressive tumor microenvironment
STING: Stimulator of interferon genes
αTim-3: anti-T cell immunoglobulin and mucin-domain containing-3 antibody
DC: dendritic cells
cDC: conventional dendritic cells
cGAMP: cyclic guanosine monophosphate-adenosine monophosphate
cGAS: cGAMP synthase
TRAF: tumor necrosis factor receptor-associated factor
TANK: TRAF-associated NF-κB activator
TBK: TANK binding kinase
IRF3: interferon regulatory factor 3
PD-1: programmed death-1
IFN-I: type I interferons
NK: natural killer
BMDCs: Bone marrow derived dendritic cells
BMDMs: Bone marrow-derived macrophages
UCB: umbilical cord blood
HSCs: hematopoietic stem cells
HE: hematoxylin and eosin
tDLNs: tumor-draining lymph nodes
FACS: fluorescence-activated cell sorting
Th: T-helper
OVA: ovalbumin peptide
DEGs: differentially expressed genes
GO: Gene Ontology
ELISA: enzyme-linked immunosorbent assay
ELISpot: enzyme-linked immunosorbent spot
NSCLC: non-small cell lung cancer
NAC: neoadjuvant chemotherapy
NAPC: neoadjuvant pembrolizumab and chemotherapy
OS: overall survival
PFS: progression-free survival
MPR: major pathological response
mIHC: multiplex immunohistochemistry
FFPE: formalin-fixed and paraffin-embedded
TCR: T-cell receptor
CXCL: C-X-C motif ligand
TILs: tumor-infiltrating lymphocytes
CTL: cytotoxic lymphocytes
TAMs: tumor-associated macrophages
Treg: regulatory T cells
Breg: regulatory B cells
MDSCs: myeloid-derived suppressor cells
pDC: plasmacytoid DC
MHC: major histocompatibility complex
ICIs: immune checkpoint inhibitors
CTLA-4: cytotoxic T lymphocyte-associated protein 4
IT: intratumoral
CIK: cytokine induced killer
