# Supplementary material for: Blocking Tim-3 enhances the anti-tumor immunity of STING agonist ADU-S100 by unleashing CD4+ T cells through regulating type 2 conventional dendritic cells
Source: Theranostics. 2023 Sep 4;13(14):4836–57. doi: 10.7150/thno.86792 (PMC10526657; doi:10.7150/thno.86792)
Supplement: Supplementary file 1 — Supplementary figures and tables. [file thnov13p4836s1.zip › supplementary.docx]

**Supplementary**

**Figure S1**


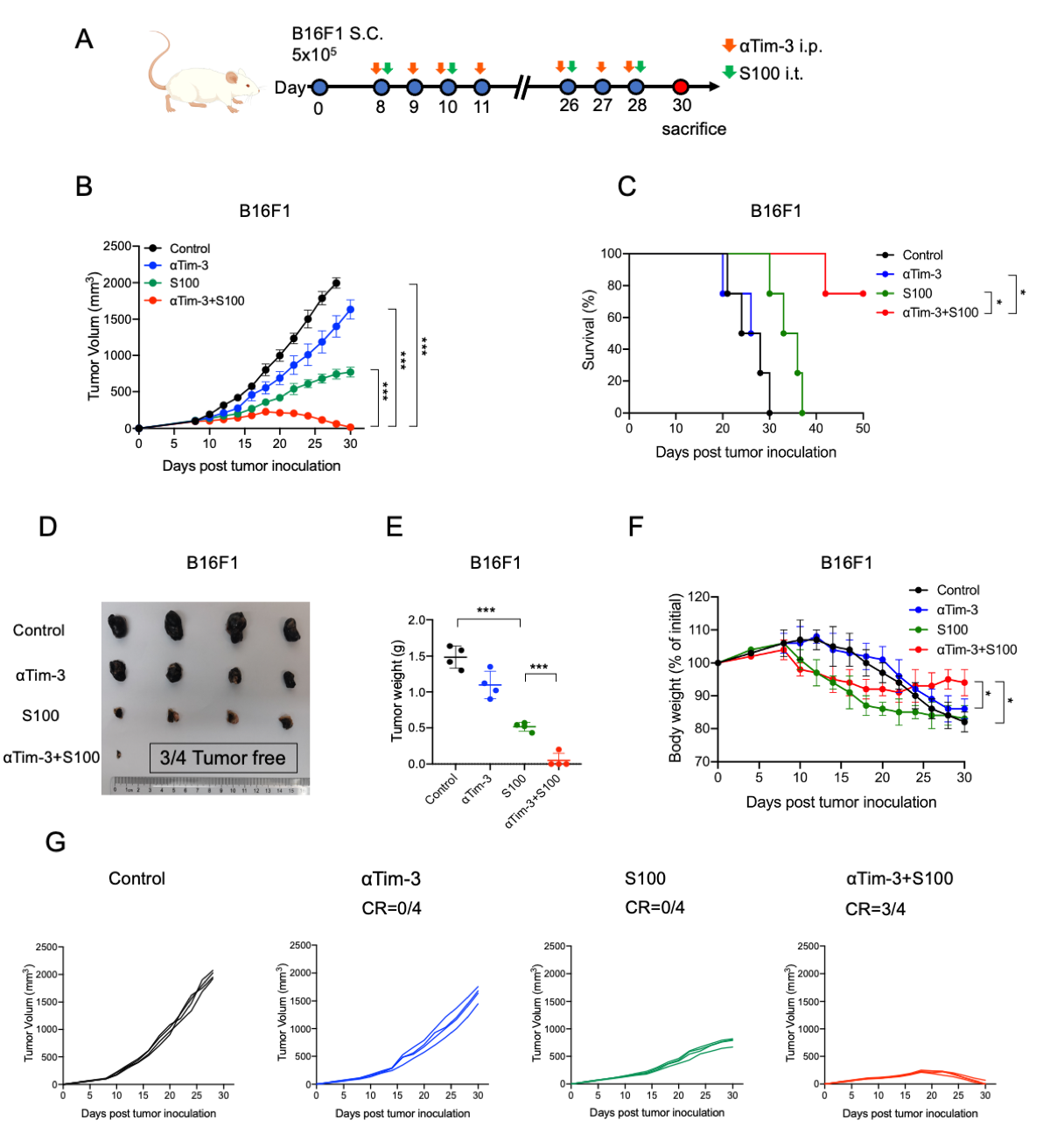


**Figure S1. αTim-3 significantly increased the antitumor response of S100 in B16F1 tumor model.** (**A**) Workflow for the B16F1 melanoma model and treatment with S100 + αTim-3. (**B)** Tumor volume, (**C**) survival, **(D)** representative images of tumors, **(E)** tumor weights, (**F**) body weight changes of mice bearing B16F1 tumors treated with S100 or αTim-3 monotherapies, as well as the combination therapy compared with control treatment (n = 4 mice per group). (**G**) Tumor growth curves of individual mice in different groups. S100, ADU-S100; αTim-3, anti-Tim-3; S.C., subcutaneous injection; i.p., intraperitoneal injection; i.t., intertumoral injection; CR, complete response. Data are presented as means ± SD. *p < 0.05; ***p < 0.01. Unpaired two-tailed Student’s t-test.

**Figure S2**


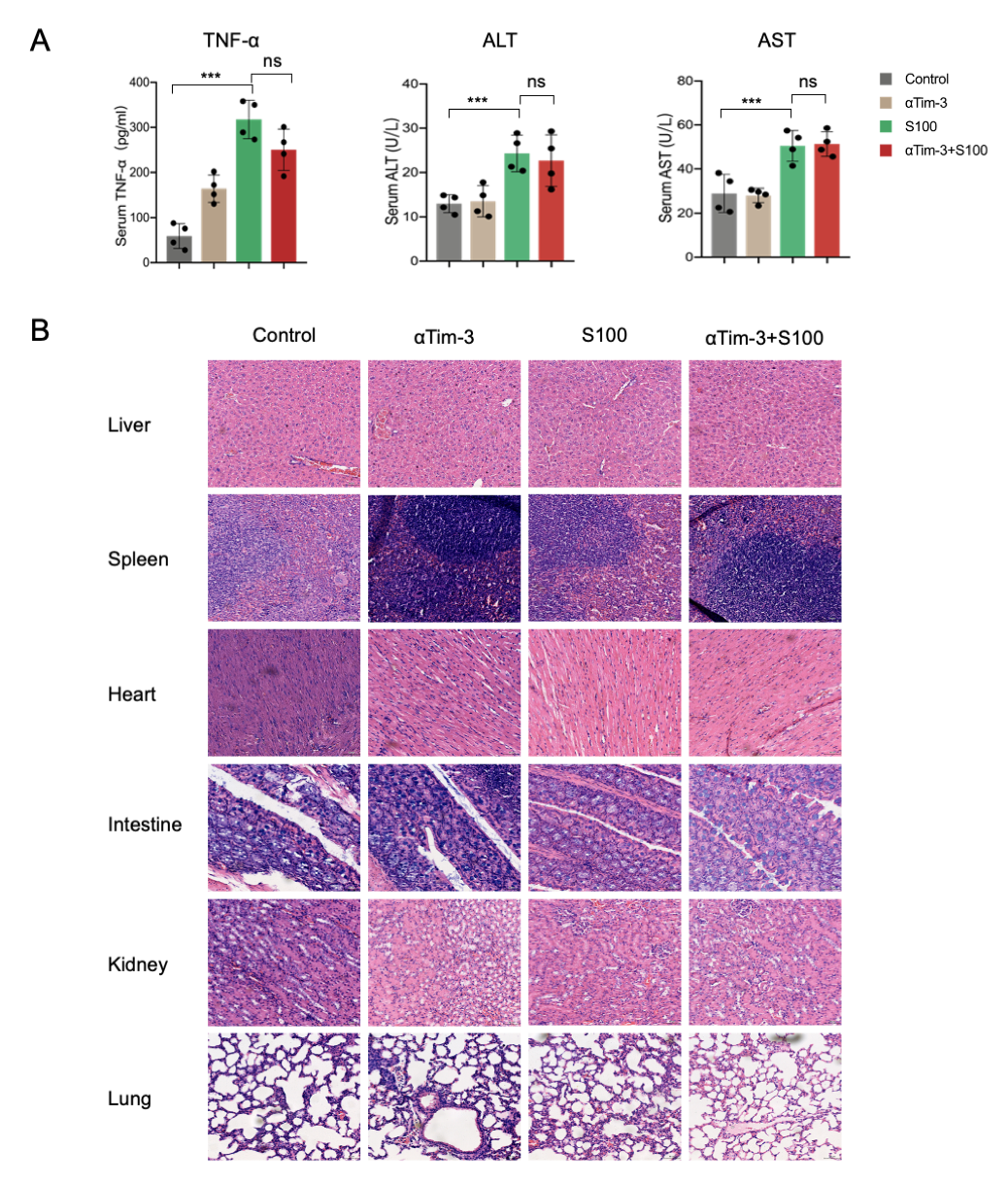


**Figure S2. Toxicities of combination treatments were acceptable.** (**A**) ELISA of inflammatory cytokine and hepatic transaminases in serum of mice bearing 4T1 tumors across different treatment groups. (**B**) Representative images of vital organ HE staining. ELISA, enzyme-linked immunosorbent assay; HE, hematoxylin and eosin; S100, ADU-S100; αTim-3, anti-Tim-3. Data are presented as means ± SD. ***p < 0.01; ns, not significant. Unpaired two-tailed Student’s t-test.

**Figure S3**


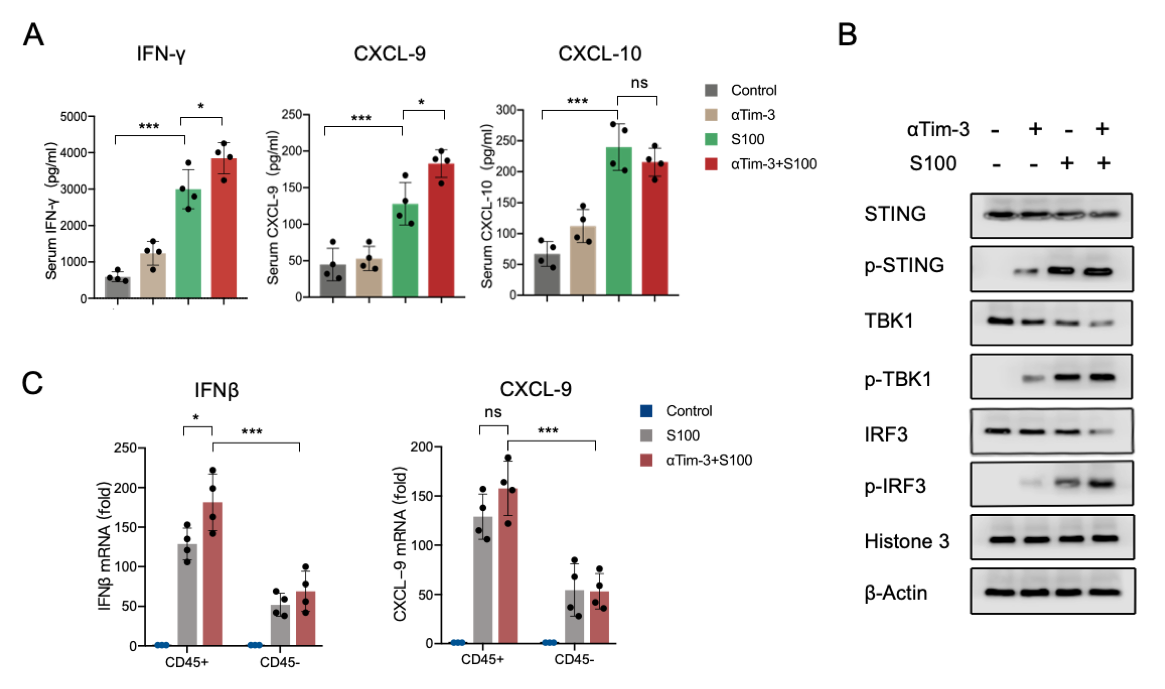


**Figure S3. STING pathway activation via combination therapy in tumor tissues**. (**A**) ELISA of IFN-γ and chemokines CXCL-9 and CXCL-10 in the serum of mice bearing 4T1 tumors across different treatment groups. (**B**) Western blot of STING activation in tumor tissues from mice in different treatment groups. (**C**) STING pathway activation via combination therapy was in host cells (CD45^+^) instead of cancer cells (CD45^-^). ELISA, enzyme-linked immunosorbent assay; S100, ADU-S100; αTim-3, anti-Tim-3; CXCL-9, C-X-C motif chemokine ligand 9. Data are presented as means ± SD. *p < 0.05; ***p < 0.01; ns, not significant. Unpaired two-tailed Student’s t-test.

**Figure S4**


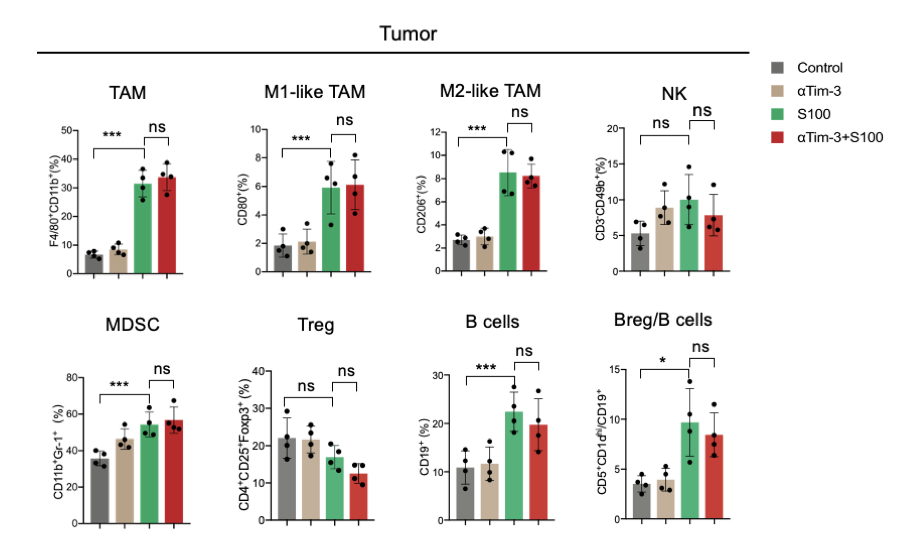


**Figure S4. Effect of combination treatment on immune cell profiles.** Quantification of tumor infiltrating TAM, including M1-like TAM and M2-TAM, NK cells, MDSC, Treg, B cells, and Breg of mice in different treatment groups. TAM, tumor-associated macrophages; MDSC, myeloid-derived suppressor cell; Breg, regulatory B cells; Treg, regulatory T cells; NK, natural killer. S100, ADU-S100; αTim-3, anti-Tim-3. Data are presented as means ± SD. ***p < 0.01; ns, not significant. Unpaired two-tailed Student’s t-test.

**Figure S5**


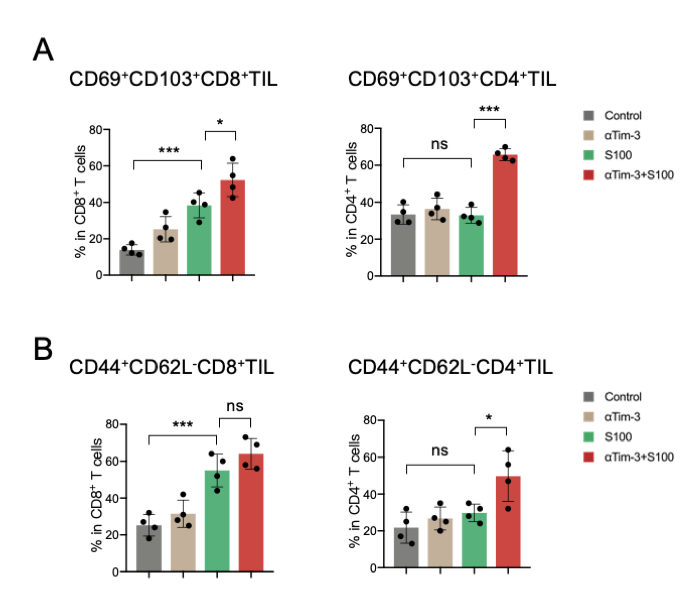


**Figure S5. Effect of combination treatment on memory T cells.** Quantification of tumor infiltrating tissue-resident memory T cells **(A)** and effector memory T cells **(B)** in both CD4^+^ and CD8^+^ T cell subsets. S100, ADU-S100; αTim-3, anti-Tim-3. Data are presented as means ± SD. *p < 0.05; ***p < 0.01. Unpaired two-tailed Student’s t-test.

**Figure S6**

**
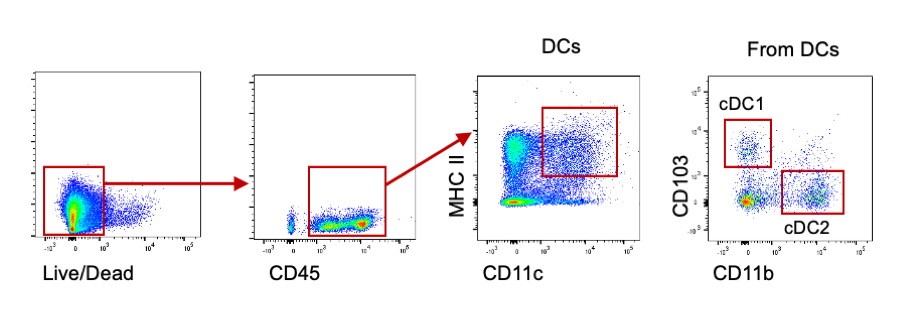
**

**Figure S6. Gating strategy for the characterization of total DC (CD11c^+^), cDC1 (CD11c^+^ MHC II^+^ CD103^+^), and cDC2 (CD11c^+^ MHC II^+^ CD11b^+^).** MHC, major histocompatibility complex; cDC, conventional dendritic cell.

**Figure S7**


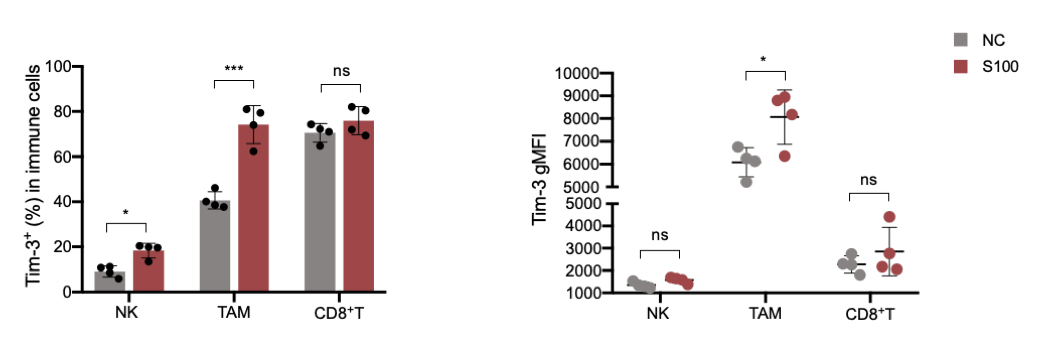


**Figure S7. S100 increased the expression of Tim-3 in NK cells and macrophages but not CD8^+^ T cells.** Expression of Tim-3 in NK cells, TAM, and CD8^+^ T cells infiltrated in tumors of mice bearing 4T1 from different treatment groups (n = 4 per group). Tumors were collected and dissected at the indicated times, the frequency of Tim-3 expressed in immune cells (left) and the gMFI of Tim-3 in immune cells (right) were analyzed using flow cytometry. TAM, tumor-associated macrophages; S100, ADU-S100; gMFI, geometric mean fluorescence intensity. Data are presented as mean ± SD. *p < 0.05; ***p < 0.01; ns, not significant. Unpaired two-tailed Student’s *t*-test.

**Figure S8**


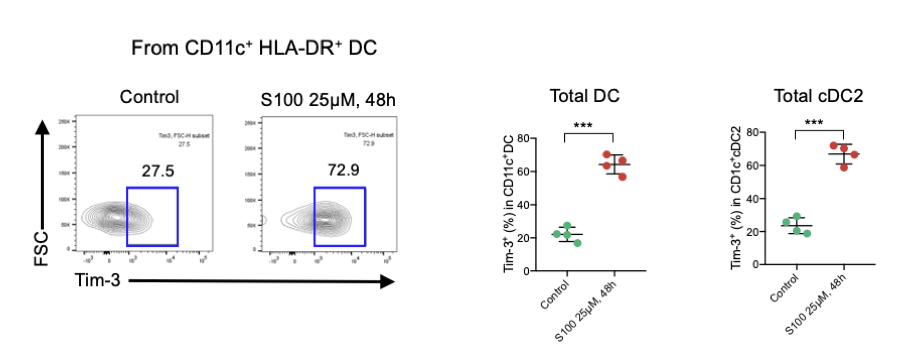


**Figure S8. S100 increased Tim-3 expression on human DCs in 25 μM.** Human DCs were generated from human stem cells, induced to maturation, and co-cultured with 25 μM S100 at 48 h. They displayed a significant up-regulation of Tim-3 both in total DCs and CD1c^+^ cDC2. S100, ADU-S100; αTim-3, anti-Tim-3, cDC, conventional dendritic cell. Data are presented as means ± SD. *p < 0.05; ***p < 0.01. Unpaired two-tailed Student’s t-test.

**Figure S9**

**
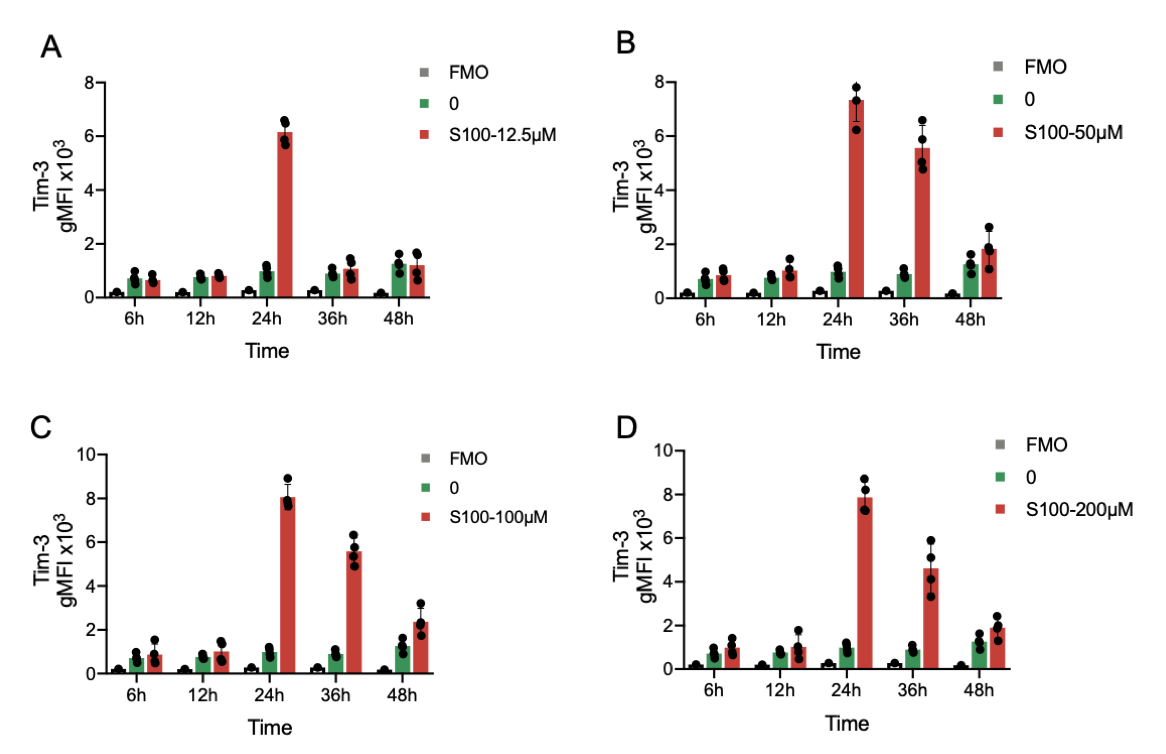
**

**Figure S9. S100 increased Tim-3 expression on BMDCs in different concentrations (12.5, 50, 100, and 200 μM).** S100, ADU-S100; BMDCs, bone marrow-derived DCs.

**Figure S10**

**
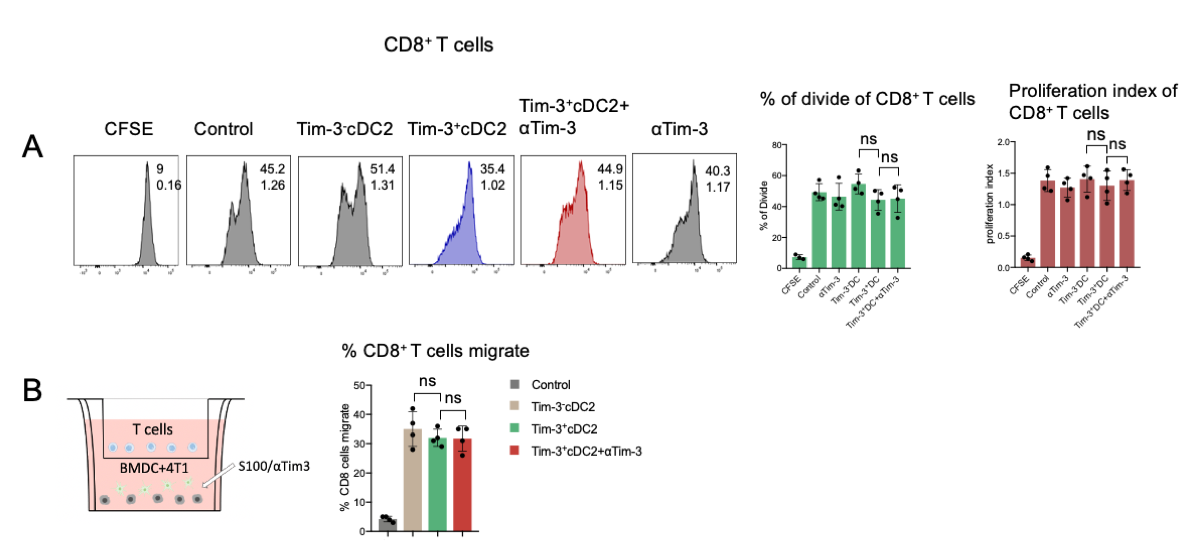
**

**Figure S10. cDC2 with high expression of Tim-3 had minimal effect on CD8^+^ T cell proliferation and migration. (A)** Representative FACS image and of the proliferation rate for CFSE-labeled CD8^+^ T cells incubated with Tim-3^-^ cDC2 or Tim-3^+^ cDC2 (with or without αTim-3), as well as the proliferation parameter percentage of divide and proliferation index. (**B**) Illustration of chemotaxis assay for CD8^+^ T cells toward Tim-3^-^ cDC2 or Tim-3^+^ cDC2 mixed tumor co-cultures (left). Quantification of CD8^+^ T cell migration in the tumor-conditioned medium in the presence of Tim-3^-^ cDC2 or Tim-3^+^ cDC2 (with or without αTim-3; right).

**Figure S11**


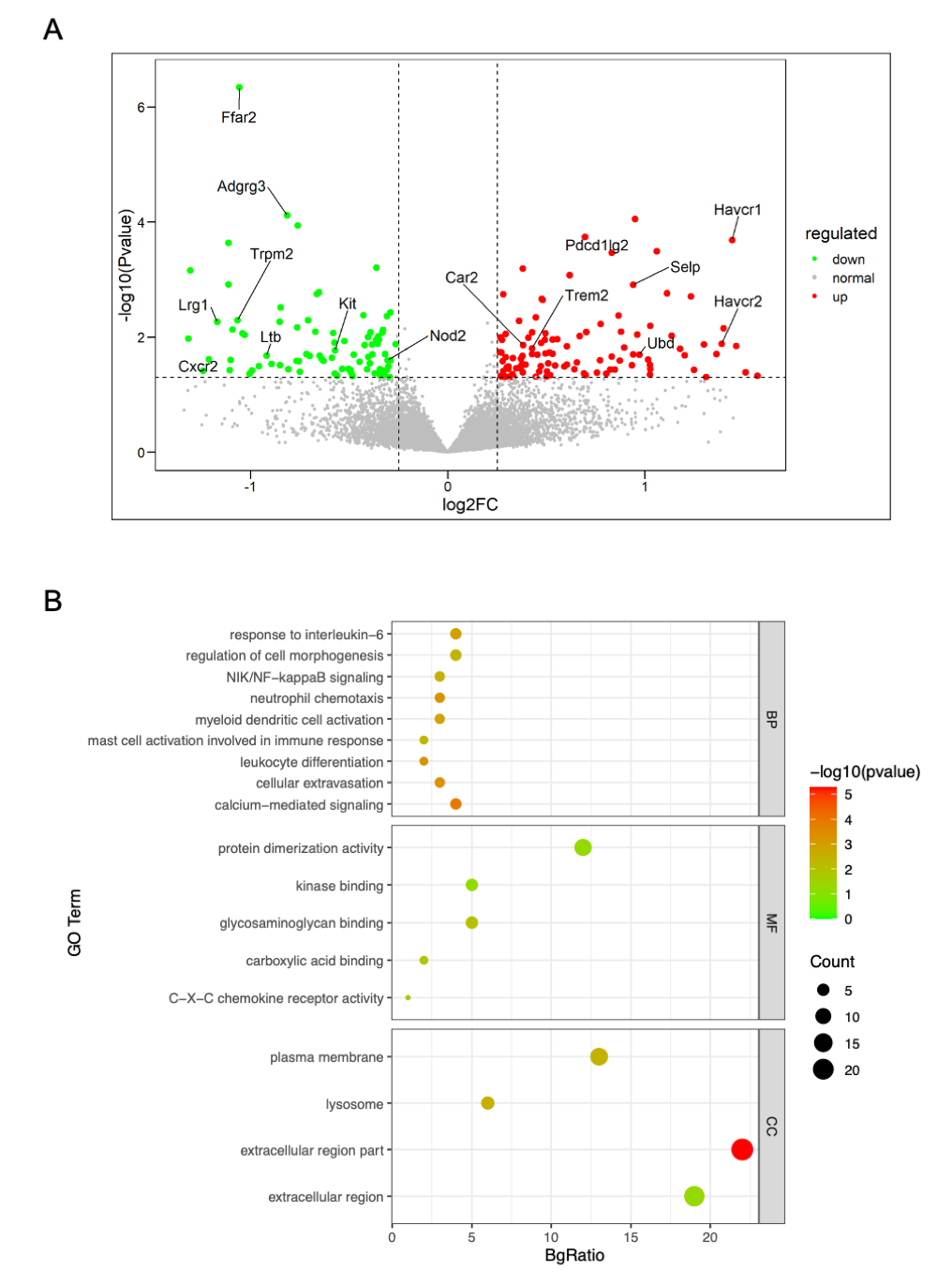


**Figure S11. (A)** Volcano plot for DEGs identified in Tim-3^+^cDC2. **(B)** Bubble plot of the GO function enrichment analysis of Tim-3^+^cDC2. DEGs, differentially expressed genes; GO, Gene Ontology.

**Figure S12**


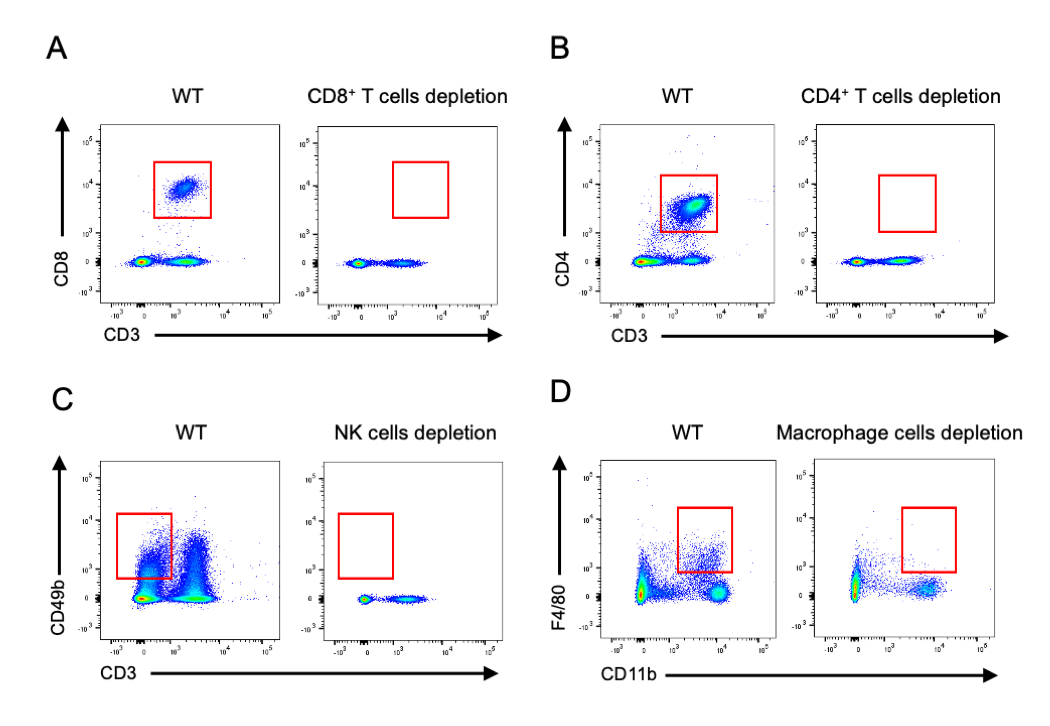


**Figure S12.** (**A**) **CD8^+^ T cell, (B) CD4^+^ T cell, (C) NK cell, or (D) macrophage depletion efficacy.** NK, natural killer. WT, wild type.

**Figure S13**

**
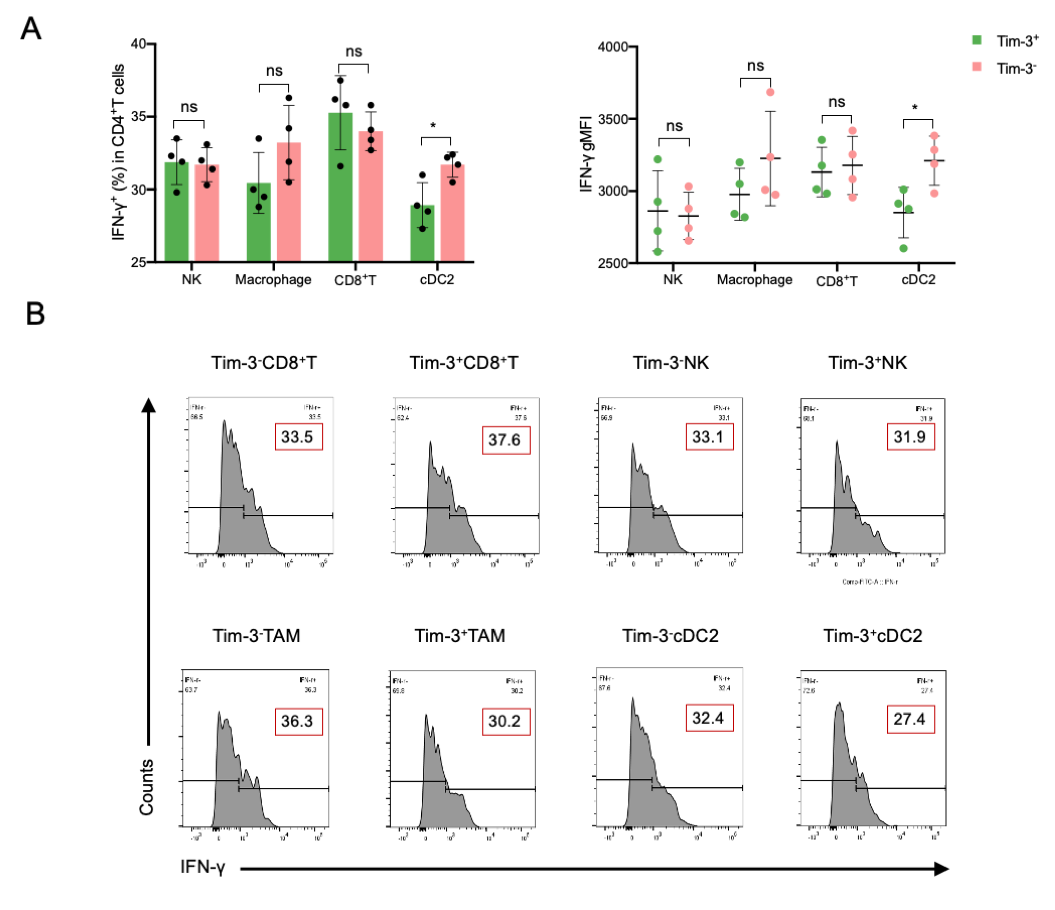
**

**Figure S13.** **Intravenous injection with Tim-3^+^cDC2 impaired the cytotoxicity of CD4^+^ T cells *in vivo* but not that of other cells.** BMDCs, BMDMs, NK cells, and CD8^+^ T cells were generated and co-cultured with 25 μM of S100, and Tim-3 expression was detected via flow cytometry. A total of 2 × 10^6^ immune cells with high or low Tim-3 expression were intravenously injected into 4T1 tumor-bearing mice one day before S100+αTim-3 treatment. The frequency of IFN-γ^+^ CD4^+^ T cells (**A)** and gMFI of IFN-γ in CD4^+^ T cells **(B**) were analyzed using flow cytometry. (**C**) Representative image of IFN-γ^+^ CD4^+^ T cells from CD4^+^ T cells primed by immune cells with high or low Tim-3 expression. BMDCs, bone marrow-derived DCs; BMDMs, bone marrow-derived macrophages; NK, natural killer; gMFI, geometric mean fluorescence intensity. Data are presented as mean ± SD. *p < 0.05; ***p < 0.01; ns, not significant as revealed by an unpaired two-tailed Student’s *t*-test.

**Figure S14**


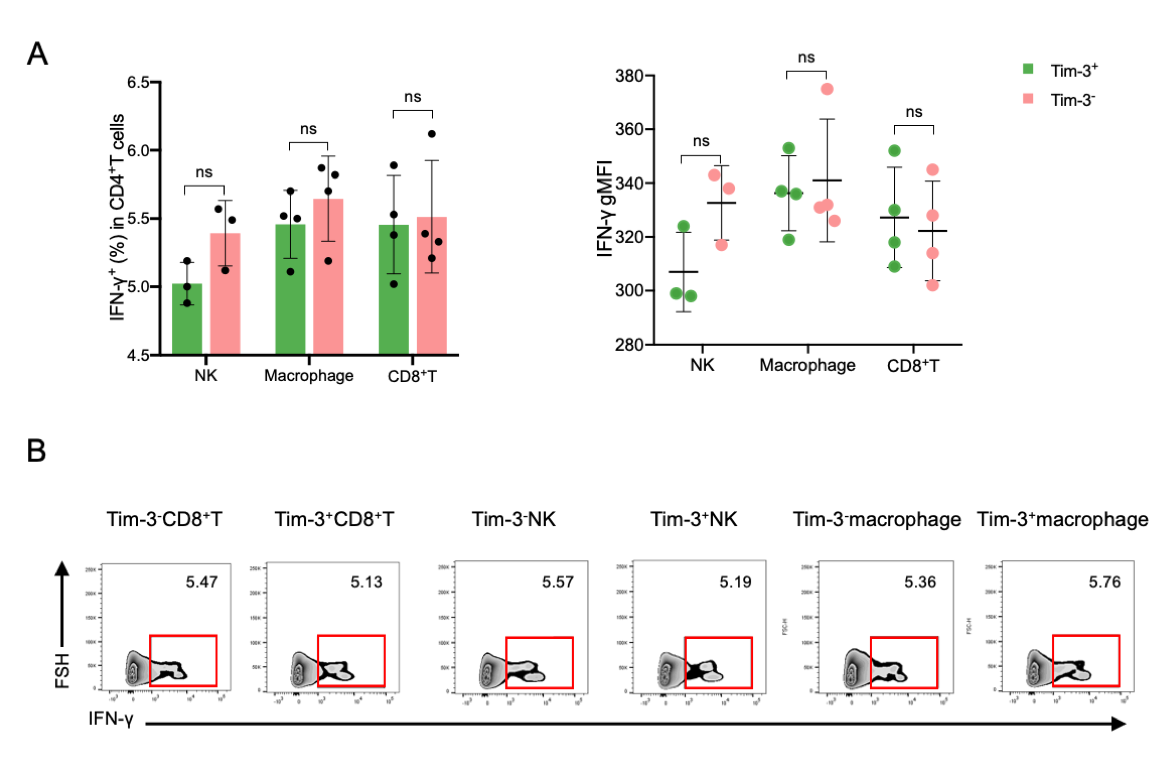


**Figure S14.** **NK cells, macrophages, and CD8^+^T cells with high Tim-3 expression have a minimal effect on CD4^+^ T cell priming *in vitro*.** BMDMs, NK cells, and CD8^+^ T cells were generated and co-cultured with S100, and Tim-3 expression was detected via flow cytometry. Immune cells with high or low Tim-3 expression were mixed with tumor cells for 24 h. The frequency of IFN-γ^+^ CD4^+^ T cells (**A)** and the gMFI of IFN-γ in CD4^+^ T cells **(B**) were analyzed using flow cytometry. **(C)** Representative image of IFN-γ^+^ CD4^+^ T cells from CD4^+^ T cells primed by immune cells with high or low Tim-3 expression. BMDMs, bone marrow-derived macrophages; NK, natural killer; gMFI, geometric mean fluorescence intensity. Data are presented as mean ± SD. *p < 0.05; ***p < 0.01; ns, not significant as revealed by an unpaired two-tailed Student’s *t*-test.

**Figure S15**


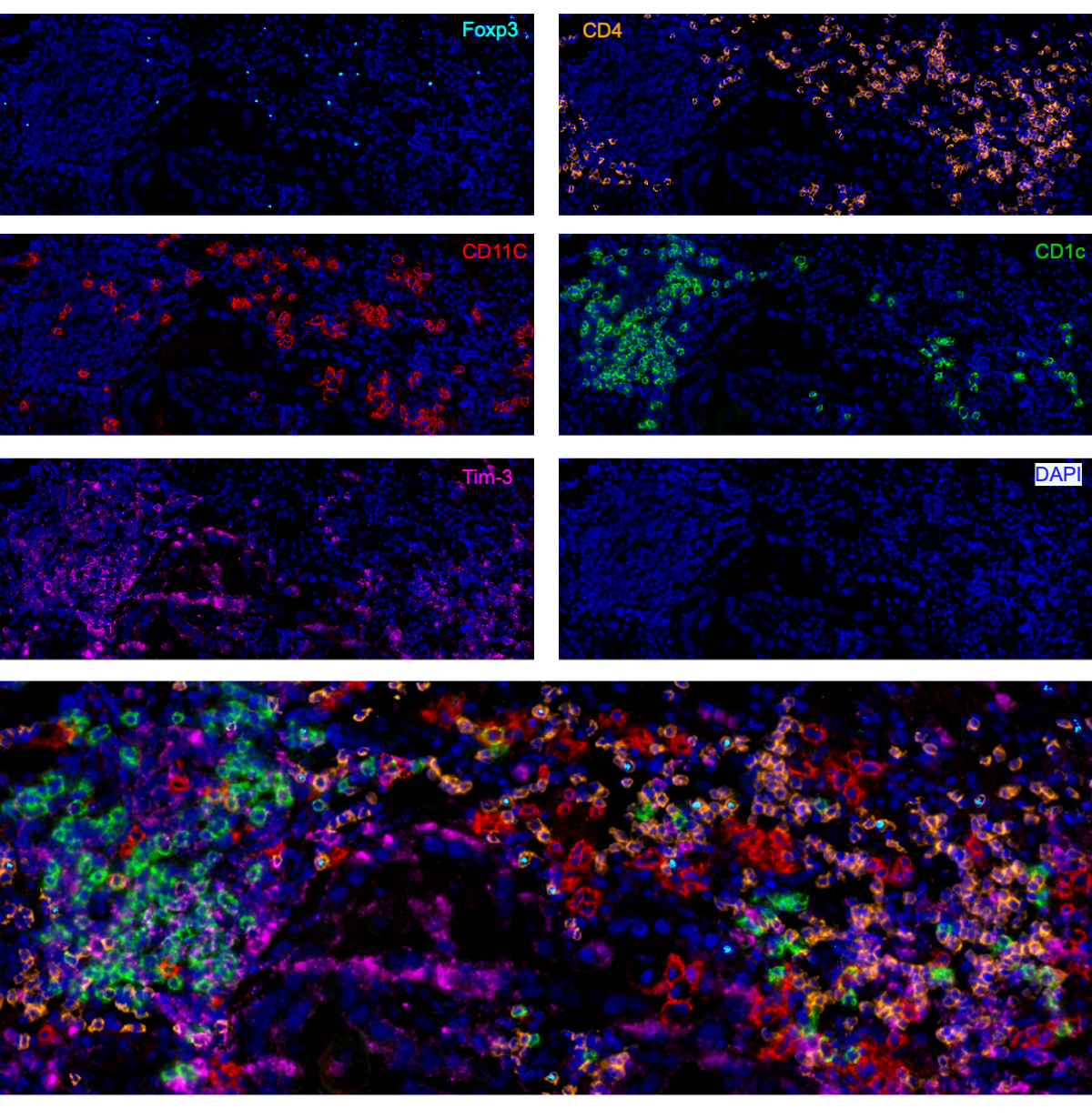


**Figure S15. Representative mIHC staining image (20×) of tumor samples in single channel.** mIHC, multiplex immunohistochemistry.

**Figure S16**

**
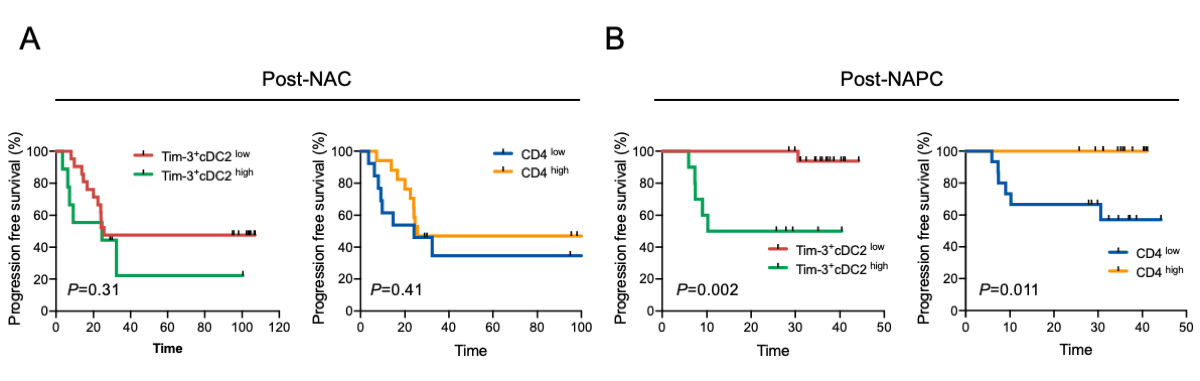
**

**Figure S16. Comparison of PFS for patients with different Tim-3^+^cDC2 or CD4^+^ T statuses treated with (A) NAC or (B) NAPC.** cDC, conventional dendritic cell; PFS, progression-free survival; NAC, neoadjuvant chemotherapy; NAPC, neoadjuvant pembrolizumab and chemotherapy. Log-rank tests were used to determine statistical significance.

**Figure S17**


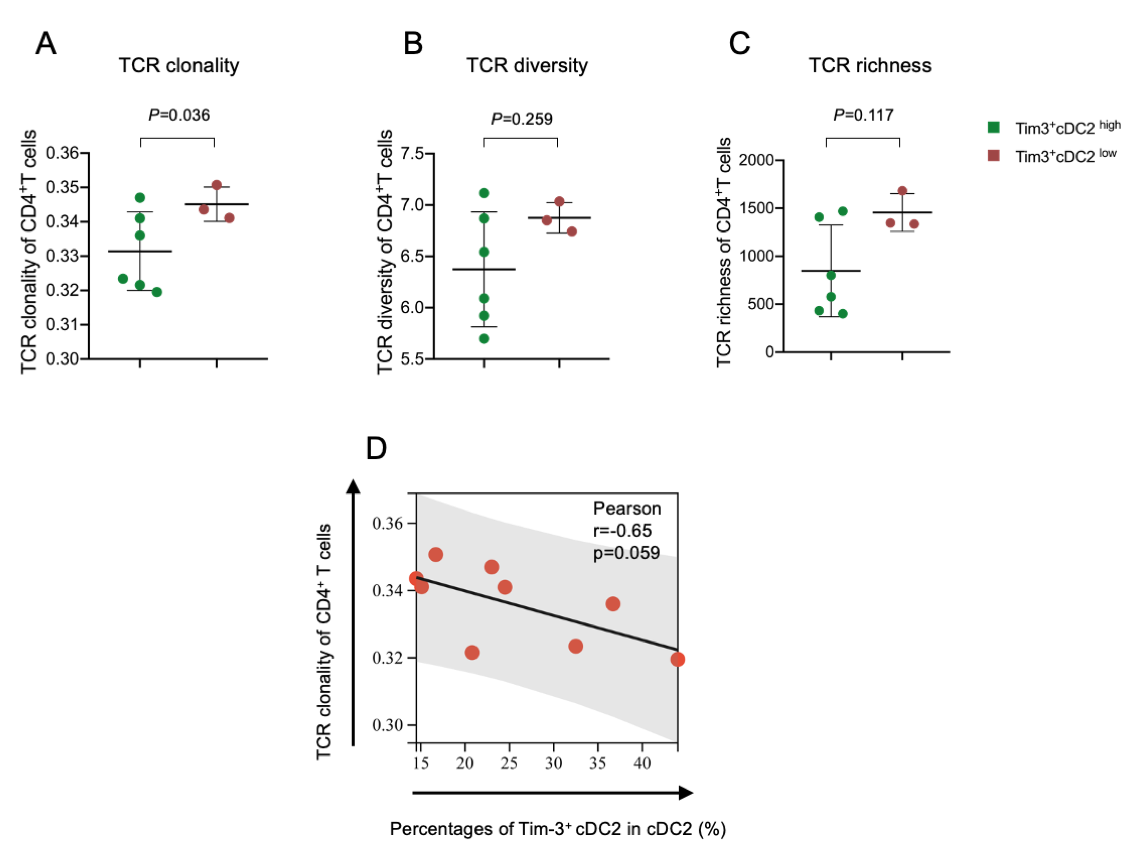


**Figure S17. Percentage of Tim-3^+^cDC2 remodel TCR of CD4^+^ T cells.** Comparison of (**A**) TCR clonality, (**B**) TCR diversity, and (**C**) TCR richness for tumor patients (n = 9) with Tim-3^+^cDC2^high^ or Tim-3^+^cDC2^low.^ (**D**) Correlation between CD4^+^ T cell clonality and Tim-3^+^cDC2 percentages. TCR, T cell receptor, cDC, conventional dendritic cell. Data are presented as means ± SD, unpaired two-tailed Student’s t-tests were used to determine statistical significance.

**Table S1**: Comparison of key differentially expressed genes associated with Tim-3^+^cDC2 and Tim-3^-^cDC2

| Gene ID | Count | | | | | | FPKM | | | | | | p | Log2FC | regulated |
| --- | --- | --- | --- | --- | --- | --- | --- | --- | --- | --- | --- | --- | --- | --- | --- |
|  | Tim-3^-^cDC2 | | | Tim-3^+^cDC2 | | | Tim-3^-^cDC2 | | | Tim-3^+^cDC2 | | |  |  |  |
| Mcemp1 | 994 | 1437 | 2974 | 848 | 948 | 1489 | 16.06 | 16.03 | 25.84 | 12.26 | 11.48 | 10.02 | 0.002 | -0.665 | down |
| Tbc1d2 | 1218 | 1853 | 2963 | 1097 | 1414 | 2290 | 6.82 | 7.18 | 8.93 | 5.49 | 5.93 | 5.34 | 0.013 | -0.357 | down |
| Cacna1d | 1465 | 1940 | 2202 | 1934 | 2091 | 5570 | 4.92 | 4.67 | 4.21 | 5.69 | 5.19 | 8.03 | 0.031 | 0.544 | up |
| Cxcr2 | 37 | 59 | 72 | 24 | 25 | 16 | 0.29 | 0.32 | 0.31 | 0.16 | 0.15 | 0.05 | 0.039 | -1.244 | down |
| Plekho1 | 4501 | 6688 | 8502 | 5794 | 6858 | 16050 | 69.12 | 69.17 | 71.50 | 77.45 | 77.56 | 95.54 | 0.029 | 0.377 | up |
| Tmem242 | 1055 | 1466 | 2369 | 1307 | 1547 | 3798 | 26.90 | 25.84 | 32.64 | 29.83 | 29.64 | 40.43 | 0.036 | 0.307 | up |
| MSTRG.1073 | 41 | 75 | 234 | 42 | 30 | 78 | 0.26 | 0.36 | 0.96 | 0.31 | 0.19 | 0.27 | 0.025 | -1.104 | down |
| Abcb10 | 125 | 165 | 425 | 93 | 133 | 256 | 0.70 | 0.64 | 1.28 | 0.46 | 0.56 | 0.60 | 0.043 | -0.575 | down |
| Car2 | 3488 | 4874 | 6495 | 4753 | 5617 | 10432 | 47.99 | 46.61 | 49.59 | 58.84 | 58.44 | 61.87 | 0.014 | 0.382 | up |
| Xpnpep3 | 352 | 467 | 825 | 179 | 349 | 665 | 1.72 | 1.38 | 1.99 | 1.76 | 1.14 | 1.29 | 0.023 | -0.589 | down |
| Tmem254 | 279 | 397 | 673 | 444 | 464 | 834 | 3.16 | 3.24 | 4.40 | 4.90 | 4.01 | 4.28 | 0.035 | 0.347 | up |
| Tnfsf15 | 2723 | 3953 | 3757 | 4918 | 5283 | 9491 | 11.29 | 11.33 | 8.41 | 18.24 | 16.42 | 16.41 | 0.006 | 0.775 | up |
| Zbtb22 | 773 | 1152 | 1837 | 733 | 886 | 1637 | 8.19 | 7.46 | 10.04 | 6.20 | 6.20 | 9.24 | 0.013 | -0.265 | down |
| Zbtb26 | 111 | 152 | 314 | 95 | 92 | 244 | 0.57 | 0.48 | 0.80 | 0.39 | 0.31 | 0.47 | 0.041 | -0.492 | down |
| Msmo1 | 645 | 920 | 1691 | 922 | 988 | 2601 | 7.72 | 7.49 | 10.89 | 9.80 | 8.81 | 12.87 | 0.034 | 0.364 | up |
| Klrb1b | 72 | 88 | 250 | 104 | 161 | 710 | 0.77 | 0.65 | 1.46 | 1.01 | 1.31 | 3.22 | 0.045 | 1.025 | up |
| Sin3a | 1523 | 2172 | 4280 | 1397 | 1703 | 3274 | 7.13 | 7.00 | 10.80 | 5.82 | 6.00 | 6.44 | 0.011 | -0.350 | down |
| Zc2hc1a | 244 | 368 | 594 | 332 | 423 | 758 | 1.79 | 1.86 | 2.34 | 2.17 | 2.32 | 2.31 | 0.047 | 0.273 | up |
| Ceacam10 | 34 | 52 | 127 | 27 | 35 | 50 | 0.76 | 0.87 | 1.66 | 0.54 | 0.60 | 0.48 | 0.031 | -0.852 | down |
| Mmp2 | 1284 | 1854 | 5079 | 1034 | 1206 | 1202 | 10.08 | 10.04 | 21.53 | 7.32 | 7.13 | 3.95 | 0.009 | -1.033 | down |
| Rgs9 | 41 | 34 | 144 | 88 | 85 | 472 | 0.40 | 0.25 | 0.91 | 0.87 | 0.84 | 2.39 | 0.020 | 1.363 | up |
| Gkap1 | 357 | 554 | 763 | 551 | 538 | 1233 | 5.94 | 6.29 | 7.24 | 8.24 | 6.59 | 9.10 | 0.024 | 0.371 | up |
| A530064-  D06Rik | 538 | 832 | 877 | 749 | 876 | 3143 | 7.06 | 6.95 | 6.39 | 8.29 | 8.08 | 16.19 | 0.025 | 0.772 | up |
| Map4k2 | 1854 | 2612 | 5311 | 1604 | 1970 | 4098 | 9.96 | 9.94 | 15.82 | 8.04 | 7.91 | 9.73 | 0.008 | -0.394 | down |
| Chst10 | 77 | 125 | 492 | 59 | 99 | 151 | 0.37 | 0.68 | 2.09 | 0.40 | 0.57 | 0.52 | 0.044 | -1.007 | down |
| Zfp748 | 215 | 289 | 433 | 172 | 209 | 364 | 0.98 | 0.91 | 1.06 | 0.70 | 0.71 | 0.69 | 0.022 | -0.404 | down |
| Jrk | 41 | 77 | 118 | 29 | 32 | 77 | 0.17 | 0.22 | 0.26 | 0.11 | 0.10 | 0.13 | 0.005 | -0.854 | down |
| Serpinb6a | 3304 | 4553 | 5024 | 4328 | 5222 | 10035 | 53.06 | 50.18 | 43.44 | 62.33 | 62.37 | 67.53 | 0.049 | 0.446 | up |
| Nlgn2 | 2938 | 4237 | 4767 | 3471 | 4674 | 11820 | 13.09 | 13.36 | 11.60 | 13.78 | 16.65 | 22.74 | 0.048 | 0.502 | up |
| Atp6v0e2 | 702 | 1078 | 1695 | 891 | 1139 | 2754 | 9.47 | 10.09 | 12.30 | 10.77 | 11.54 | 15.48 | 0.045 | 0.328 | up |
| Il12b | 6552 | 9236 | 9657 | 15472 | 16410 | 23829 | 84.95 | 82.61 | 67.37 | 179.58 | 159.62 | 128.68 | 0.000 | 1.059 | up |
| Slc32a1 | 303 | 552 | 1031 | 180 | 204 | 115 | 2.61 | 3.29 | 4.79 | 1.39 | 1.31 | 0.41 | 0.002 | -1.640 | down |
| B3galnt1 | 740 | 1074 | 1459 | 1162 | 1304 | 4707 | 8.80 | 8.82 | 9.34 | 12.37 | 11.64 | 23.33 | 0.004 | 0.865 | up |
| Slc13a3 | 26 | 30 | 126 | 45 | 72 | 537 | 0.18 | 0.14 | 0.46 | 0.27 | 0.37 | 1.54 | 0.039 | 1.575 | up |
| Taf2 | 825 | 1193 | 2201 | 807 | 953 | 1748 | 4.10 | 4.16 | 6.21 | 3.65 | 3.80 | 3.83 | 0.025 | -0.290 | down |
| Tbl3 | 428 | 614 | 992 | 419 | 446 | 785 | 1.97 | 1.96 | 2.46 | 1.73 | 1.54 | 1.51 | 0.038 | -0.331 | down |
| Fasn | 1017 | 1460 | 2433 | 805 | 1167 | 2201 | 2.73 | 2.48 | 3.41 | 1.73 | 2.14 | 2.26 | 0.020 | -0.319 | down |
| Timm21 | 144 | 211 | 440 | 148 | 142 | 264 | 1.53 | 1.45 | 2.37 | 1.32 | 1.06 | 1.11 | 0.042 | -0.492 | down |
| Rprm | 30 | 39 | 98 | 78 | 70 | 158 | 0.49 | 0.44 | 0.86 | 1.15 | 0.87 | 1.08 | 0.008 | 0.878 | up |
| Mafb | 965 | 1511 | 1786 | 1404 | 1628 | 3664 | 6.92 | 7.48 | 6.89 | 9.01 | 8.76 | 10.95 | 0.009 | 0.495 | up |
| Pdcd1lg2 | 1150 | 1616 | 2390 | 1797 | 2265 | 6553 | 16.69 | 16.26 | 18.70 | 23.41 | 24.78 | 39.89 | 0.000 | 0.830 | up |
| Ccne1 | 315 | 510 | 716 | 449 | 591 | 972 | 3.82 | 4.29 | 4.68 | 4.89 | 5.49 | 4.92 | 0.049 | 0.332 | up |
| Slc7a6 | 534 | 702 | 1415 | 446 | 506 | 1094 | 3.60 | 3.23 | 5.18 | 2.65 | 2.56 | 3.07 | 0.004 | -0.430 | down |
| Tnfsf9 | 425 | 587 | 997 | 521 | 681 | 1651 | 7.95 | 7.58 | 10.03 | 8.72 | 9.55 | 12.86 | 0.035 | 0.368 | up |
| Ubd | 71 | 110 | 171 | 107 | 143 | 589 | 1.82 | 1.96 | 2.37 | 2.47 | 2.77 | 6.36 | 0.020 | 0.971 | up |
| Kcnj16 | 75 | 96 | 321 | 182 | 202 | 470 | 0.50 | 0.44 | 1.15 | 1.08 | 1.00 | 1.30 | 0.022 | 0.830 | up |
| Mucl1 | 136 | 195 | 244 | 415 | 445 | 1272 | 4.31 | 4.29 | 4.17 | 11.82 | 10.62 | 16.87 | 0.000 | 1.684 | up |
| Slc39a8 | 89 | 145 | 264 | 157 | 160 | 709 | 0.62 | 0.72 | 1.01 | 1.01 | 0.85 | 2.11 | 0.037 | 0.823 | up |
| Slc39a4 | 324 | 440 | 852 | 208 | 297 | 453 | 2.86 | 2.70 | 4.08 | 1.64 | 1.97 | 1.69 | 0.000 | -0.761 | down |
| Cep19 | 387 | 500 | 1037 | 588 | 640 | 1666 | 5.45 | 4.87 | 7.87 | 7.45 | 6.76 | 9.74 | 0.011 | 0.493 | up |
| Rfx5 | 650 | 814 | 1741 | 604 | 677 | 1283 | 3.92 | 3.38 | 5.71 | 3.43 | 3.15 | 3.27 | 0.048 | -0.337 | down |
| MSTRG.14087 | 64 | 77 | 157 | 97 | 98 | 387 | 0.94 | 0.77 | 1.26 | 1.28 | 1.09 | 2.40 | 0.042 | 0.756 | up |
| Ece1 | 2840 | 3853 | 5808 | 3561 | 4041 | 8610 | 14.69 | 14.17 | 16.73 | 16.95 | 15.95 | 19.09 | 0.018 | 0.263 | up |
| Ffar2 | 160 | 212 | 311 | 85 | 91 | 171 | 2.52 | 2.08 | 2.28 | 1.13 | 1.02 | 0.99 | 0.000 | -1.059 | down |
| Mgst2 | 600 | 863 | 1240 | 786 | 1004 | 1938 | 24.83 | 24.90 | 28.38 | 29.57 | 31.19 | 34.02 | 0.005 | 0.361 | up |
| Tmeff1 | 413 | 594 | 555 | 595 | 663 | 1640 | 4.86 | 4.51 | 3.46 | 5.70 | 5.39 | 7.31 | 0.028 | 0.654 | up |
| Col18a1 | 55 | 97 | 122 | 78 | 125 | 397 | 0.26 | 0.32 | 0.31 | 0.33 | 0.45 | 0.79 | 0.026 | 0.872 | up |
| MSTRG.19311 | 42 | 67 | 227 | 29 | 25 | 58 | 2.61 | 2.91 | 7.67 | 1.60 | 1.14 | 1.51 | 0.002 | -1.473 | down |
| Tppp3 | 69 | 118 | 175 | 153 | 139 | 265 | 1.40 | 1.76 | 1.89 | 3.02 | 2.26 | 2.29 | 0.014 | 0.605 | up |
| Svbp | 587 | 890 | 1146 | 867 | 1015 | 1692 | 19.21 | 20.16 | 20.17 | 25.26 | 24.99 | 23.13 | 0.041 | 0.380 | up |
| Lxn | 1078 | 1509 | 2158 | 1461 | 1557 | 5892 | 23.38 | 22.61 | 25.19 | 28.38 | 25.34 | 53.26 | 0.037 | 0.642 | up |
| MSTRG.1192 | 132 | 137 | 472 | 312 | 353 | 1198 | 0.62 | 0.43 | 1.33 | 1.36 | 1.25 | 2.61 | 0.002 | 1.233 | up |
| Gldc | 21 | 56 | 123 | 57 | 84 | 400 | 0.13 | 0.25 | 0.42 | 0.33 | 0.40 | 1.07 | 0.037 | 1.249 | up |
| Kcnh7 | 92 | 124 | 271 | 90 | 83 | 148 | 0.17 | 0.15 | 0.26 | 0.15 | 0.11 | 0.11 | 0.047 | -0.562 | down |
| Serinc2 | 82 | 118 | 388 | 124 | 175 | 1926 | 1.04 | 1.04 | 2.67 | 1.42 | 1.68 | 10.44 | 0.047 | 1.570 | up |
| Pars2 | 126 | 156 | 229 | 82 | 105 | 178 | 1.05 | 1.06 | 1.41 | 0.82 | 0.91 | 0.71 | 0.012 | -0.577 | down |
| Man2a2 | 1911 | 2947 | 5088 | 1731 | 2043 | 4304 | 7.48 | 7.90 | 10.73 | 6.09 | 5.99 | 7.14 | 0.001 | -0.363 | down |
| Zfp955b | 341 | 454 | 860 | 308 | 349 | 634 | 1.42 | 1.30 | 1.93 | 1.15 | 1.09 | 1.11 | 0.013 | -0.385 | down |
| Tmem9 | 530 | 627 | 814 | 651 | 769 | 1679 | 7.13 | 5.78 | 5.86 | 7.78 | 7.70 | 9.34 | 0.032 | 0.472 | up |
| Rnf144a | 74 | 99 | 211 | 54 | 63 | 122 | 0.35 | 0.33 | 0.54 | 0.24 | 0.23 | 0.24 | 0.005 | -0.709 | down |
| MSTRG.5827 | 71 | 101 | 117 | 22 | 20 | 120 | 3.59 | 3.52 | 3.20 | 0.98 | 0.73 | 2.53 | 0.024 | -1.214 | down |
| Rab37 | 65 | 105 | 187 | 52 | 56 | 87 | 0.69 | 0.79 | 1.08 | 0.49 | 0.44 | 0.39 | 0.003 | -0.849 | down |
| Bin1 | 1673 | 2369 | 4216 | 2160 | 2583 | 6204 | 21.66 | 21.22 | 30.40 | 24.96 | 24.69 | 33.21 | 0.034 | 0.298 | up |
| Ripor2 | 115 | 172 | 536 | 80 | 75 | 153 | 0.65 | 0.64 | 1.53 | 0.37 | 0.26 | 0.35 | 0.001 | -1.309 | down |
| Ttc9 | 139 | 181 | 448 | 204 | 302 | 1195 | 1.01 | 0.83 | 1.60 | 1.05 | 1.33 | 3.60 | 0.031 | 0.934 | up |
| Ocstamp | 34 | 46 | 199 | 75 | 98 | 756 | 0.22 | 0.21 | 0.70 | 0.44 | 0.48 | 2.08 | 0.041 | 1.510 | up |
| Fgf23 | 72 | 102 | 249 | 155 | 159 | 587 | 0.95 | 0.93 | 1.78 | 1.85 | 1.58 | 3.25 | 0.009 | 0.961 | up |
| Grtp1 | 112 | 130 | 259 | 154 | 180 | 395 | 1.11 | 0.92 | 1.59 | 1.38 | 1.29 | 1.91 | 0.020 | 0.454 | up |
| Cbr1 | 346 | 569 | 800 | 512 | 648 | 1430 | 6.77 | 7.63 | 8.63 | 8.93 | 9.48 | 11.64 | 0.002 | 0.476 | up |
| Uqcrfs1 | 1749 | 2498 | 3569 | 2222 | 2563 | 6085 | 30.97 | 30.53 | 33.99 | 35.21 | 34.04 | 44.88 | 0.023 | 0.329 | up |
| Nod2 | 1352 | 1997 | 3744 | 1336 | 1583 | 2870 | 7.01 | 7.15 | 10.44 | 6.19 | 6.16 | 6.20 | 0.025 | -0.306 | down |
| Fblim1 | 245 | 397 | 497 | 355 | 398 | 1277 | 1.92 | 2.23 | 2.15 | 2.62 | 2.40 | 4.36 | 0.030 | 0.605 | up |
| Arfgef2 | 2658 | 3889 | 6624 | 2473 | 3054 | 5554 | 7.34 | 7.41 | 9.88 | 6.09 | 6.31 | 6.41 | 0.004 | -0.292 | down |
| Ccdc57 | 221 | 299 | 533 | 206 | 210 | 415 | 1.67 | 1.60 | 2.15 | 1.34 | 1.15 | 1.30 | 0.020 | -0.384 | down |
| Plet1 | 3083 | 4545 | 6599 | 4400 | 5565 | 32269 | 44.49 | 45.24 | 51.13 | 56.43 | 59.91 | 193.84 | 0.016 | 1.178 | up |
| 0610030E20Rik | 693 | 992 | 1679 | 605 | 776 | 1454 | 3.63 | 3.59 | 4.73 | 2.83 | 3.05 | 3.17 | 0.004 | -0.310 | down |
| Armc9 | 98 | 170 | 306 | 89 | 98 | 204 | 0.66 | 1.15 | 1.50 | 0.63 | 0.71 | 0.85 | 0.008 | -0.583 | down |
| Cd36 | 4953 | 6920 | 10346 | 8014 | 8584 | 24023 | 43.26 | 41.00 | 46.19 | 61.94 | 56.41 | 84.50 | 0.000 | 0.695 | up |
| Pmepa1 | 810 | 1280 | 1661 | 1086 | 1386 | 3775 | 4.44 | 4.82 | 4.83 | 5.26 | 5.58 | 8.51 | 0.019 | 0.532 | up |
| Dpm1 | 92 | 158 | 592 | 166 | 893 | 772 | 1.74 | 2.03 | 6.20 | 2.68 | 8.32 | 6.41 | 0.049 | 1.312 | up |
| Selplg | 5924 | 8842 | 14113 | 7696 | 9631 | 20058 | 78.26 | 80.71 | 101.28 | 90.68 | 95.47 | 110.59 | 0.002 | 0.280 | up |
| Col6a6 | 77 | 96 | 350 | 48 | 68 | 61 | 0.21 | 0.18 | 0.52 | 0.12 | 0.14 | 0.07 | 0.011 | -1.318 | down |
| Creb3l1 | 106 | 131 | 200 | 135 | 158 | 407 | 0.98 | 0.84 | 1.00 | 1.12 | 1.10 | 1.57 | 0.029 | 0.510 | up |
| Tstd3 | 357 | 550 | 851 | 614 | 589 | 1206 | 5.42 | 5.77 | 6.95 | 8.35 | 6.71 | 7.63 | 0.010 | 0.408 | up |
| Gm45837 | 753 | 841 | 1237 | 438 | 513 | 1371 | 4.39 | 3.38 | 3.88 | 2.28 | 2.24 | 3.33 | 0.036 | -0.506 | down |
| Abca13 | 115 | 200 | 288 | 95 | 82 | 95 | 0.22 | 0.23 | 0.27 | 0.15 | 0.11 | 0.06 | 0.009 | -1.043 | down |
| Nipal1 | 27 | 56 | 125 | 100 | 69 | 398 | 0.15 | 0.22 | 0.39 | 0.51 | 0.30 | 0.95 | 0.013 | 1.300 | up |
| F13a1 | 256 | 392 | 614 | 205 | 248 | 575 | 1.56 | 1.65 | 2.06 | 1.13 | 1.14 | 1.51 | 0.010 | -0.406 | down |
| Txndc15 | 1047 | 1465 | 2257 | 1401 | 1517 | 3232 | 16.54 | 15.98 | 19.19 | 19.81 | 17.98 | 21.28 | 0.011 | 0.274 | up |
| Nrn1 | 81 | 89 | 191 | 123 | 122 | 658 | 1.21 | 0.91 | 1.55 | 1.64 | 1.37 | 4.11 | 0.031 | 1.025 | up |
| Pfdn1 | 737 | 1152 | 1790 | 961 | 1195 | 2790 | 16.01 | 17.31 | 20.97 | 18.71 | 19.49 | 25.30 | 0.032 | 0.308 | up |
| Gm26992 | 444 | 614 | 1281 | 355 | 446 | 1060 | 22.87 | 21.84 | 35.53 | 16.37 | 17.22 | 22.76 | 0.036 | -0.410 | down |
| Cox6c | 2109 | 3005 | 5539 | 2703 | 3409 | 7657 | 98.88 | 98.65 | 140.27 | 113.72 | 121.44 | 151.69 | 0.039 | 0.281 | up |
| Cacnb4 | 99 | 141 | 193 | 192 | 196 | 557 | 0.29 | 0.28 | 0.32 | 0.50 | 0.43 | 0.68 | 0.000 | 0.950 | up |
| Tmc7 | 61 | 92 | 290 | 45 | 50 | 101 | 0.32 | 0.33 | 0.83 | 0.21 | 0.20 | 0.22 | 0.007 | -1.093 | down |
| Pfkfb2 | 248 | 385 | 628 | 223 | 274 | 533 | 2.06 | 2.02 | 3.16 | 1.28 | 1.57 | 2.08 | 0.010 | -0.354 | down |
| MSTRG.10768 | 67 | 114 | 197 | 134 | 150 | 708 | 0.83 | 0.89 | 1.31 | 1.55 | 1.49 | 4.04 | 0.009 | 1.136 | up |
| Cd177 | 70 | 116 | 92 | 49 | 36 | 45 | 0.62 | 0.71 | 0.44 | 0.38 | 0.24 | 0.16 | 0.038 | -1.107 | down |
| Mast4 | 206 | 320 | 500 | 284 | 328 | 696 | 0.47 | 0.51 | 1.06 | 0.64 | 0.56 | 1.08 | 0.048 | 0.268 | up |
| Nectin2 | 1086 | 1553 | 2741 | 1449 | 2004 | 4455 | 11.72 | 11.68 | 15.37 | 13.77 | 16.17 | 20.23 | 0.005 | 0.446 | up |
| Siglece | 1282 | 1819 | 2776 | 996 | 1126 | 966 | 17.01 | 16.49 | 19.67 | 11.72 | 11.13 | 5.27 | 0.036 | -0.809 | down |
| Ccnd3 | 2001 | 3050 | 4803 | 2570 | 3104 | 7943 | 25.44 | 28.72 | 36.00 | 29.62 | 32.62 | 47.98 | 0.049 | 0.324 | up |
| Zfp940 | 42 | 57 | 123 | 23 | 32 | 88 | 0.37 | 0.35 | 0.63 | 0.18 | 0.32 | 0.40 | 0.040 | -0.750 | down |
| Bmpr1a | 724 | 929 | 1597 | 609 | 661 | 1493 | 3.02 | 2.63 | 3.53 | 2.33 | 2.03 | 2.57 | 0.008 | -0.334 | down |
| Areg | 33 | 45 | 75 | 55 | 59 | 231 | 0.65 | 0.61 | 0.79 | 0.96 | 0.88 | 1.91 | 0.020 | 0.939 | up |
| Arsg | 170 | 199 | 560 | 111 | 119 | 166 | 1.25 | 1.11 | 2.32 | 0.74 | 0.69 | 0.56 | 0.001 | -1.114 | down |
| Arap3 | 545 | 924 | 1236 | 418 | 611 | 689 | 2.68 | 3.03 | 3.32 | 2.03 | 2.21 | 1.32 | 0.026 | -0.632 | down |
| Syne1 | 6767 | 9748 | 12617 | 5620 | 6605 | 8883 | 6.02 | 6.01 | 6.62 | 4.78 | 4.46 | 3.54 | 0.048 | -0.485 | down |
| Ccdc80 | 223 | 356 | 522 | 387 | 441 | 1902 | 1.38 | 1.48 | 1.72 | 2.13 | 2.08 | 4.91 | 0.006 | 1.027 | up |
| Lars2 | 4656 | 7854 | 11995 | 10288 | 8940 | 16416 | 29.90 | 34.81 | 41.48 | 57.12 | 41.58 | 42.46 | 0.011 | 0.556 | up |
| Il22 | 117 | 136 | 240 | 320 | 269 | 606 | 2.53 | 2.03 | 2.80 | 6.22 | 4.39 | 5.48 | 0.000 | 1.212 | up |
| Coch | 241 | 332 | 388 | 329 | 403 | 1513 | 2.29 | 2.22 | 1.89 | 2.81 | 2.72 | 6.09 | 0.015 | 0.895 | up |
| Uggt1 | 2448 | 3276 | 7031 | 2259 | 2597 | 5623 | 7.00 | 6.39 | 11.14 | 5.84 | 6.14 | 6.74 | 0.046 | -0.324 | down |
| Sned1 | 156 | 210 | 337 | 106 | 148 | 148 | 0.41 | 0.38 | 0.48 | 0.26 | 0.30 | 0.17 | 0.026 | -0.756 | down |
| Lamc2 | 26 | 26 | 83 | 54 | 52 | 145 | 0.13 | 0.08 | 0.21 | 0.22 | 0.18 | 0.32 | 0.037 | 0.847 | up |
| Itgb8 | 151 | 189 | 388 | 245 | 214 | 1039 | 0.43 | 0.37 | 0.60 | 0.63 | 0.46 | 1.24 | 0.043 | 0.804 | up |
| Prom1 | 710 | 1079 | 2131 | 476 | 553 | 636 | 4.89 | 5.02 | 7.63 | 2.81 | 2.78 | 1.76 | 0.000 | -1.115 | down |
| Vcam1 | 474 | 644 | 1084 | 725 | 723 | 4177 | 3.50 | 3.28 | 4.68 | 4.64 | 3.88 | 14.32 | 0.025 | 1.016 | up |
| Ksr1 | 783 | 1206 | 2446 | 732 | 840 | 1795 | 4.05 | 4.62 | 7.29 | 3.19 | 3.13 | 4.19 | 0.014 | -0.424 | down |
| Cd109 | 349 | 501 | 836 | 478 | 581 | 1649 | 1.43 | 1.42 | 1.85 | 1.76 | 1.79 | 2.83 | 0.018 | 0.516 | up |
| Rasd2 | 56 | 92 | 332 | 12 | 40 | 78 | 0.49 | 0.55 | 1.55 | 0.09 | 0.25 | 0.28 | 0.002 | -1.870 | down |
| Lrg1 | 88 | 136 | 142 | 53 | 50 | 60 | 1.50 | 1.60 | 1.31 | 0.81 | 0.64 | 0.43 | 0.005 | -1.172 | down |
| Ankrd52 | 2678 | 3847 | 6266 | 2414 | 2981 | 5052 | 9.76 | 9.68 | 12.30 | 7.87 | 8.15 | 7.70 | 0.007 | -0.330 | down |
| Prox2 | 65 | 118 | 192 | 43 | 71 | 111 | 1.11 | 0.99 | 1.47 | 0.31 | 0.42 | 0.58 | 0.007 | -0.765 | down |
| Fgl2 | 2531 | 3627 | 5225 | 3649 | 3814 | 11320 | 16.12 | 15.96 | 17.91 | 20.81 | 18.22 | 30.04 | 0.011 | 0.532 | up |
| Cd69 | 5067 | 7162 | 8987 | 6924 | 7435 | 17632 | 72.82 | 71.16 | 69.76 | 89.04 | 80.16 | 105.60 | 0.020 | 0.427 | up |
| Havcr1 | 32 | 32 | 95 | 92 | 87 | 285 | 0.39 | 0.29 | 0.61 | 1.02 | 0.78 | 1.46 | 0.000 | 1.443 | up |
| Havcr2 | 1209 | 1849 | 3921 | 2181 | 2571 | 18378 | 10.72 | 11.32 | 18.71 | 17.31 | 17.10 | 67.89 | 0.013 | 1.391 | up |
| Fam110c | 95 | 151 | 157 | 136 | 153 | 494 | 0.74 | 0.81 | 0.66 | 0.95 | 0.89 | 1.61 | 0.042 | 0.692 | up |
| Il16 | 253 | 378 | 992 | 215 | 272 | 516 | 1.62 | 1.96 | 3.63 | 1.42 | 1.52 | 1.47 | 0.021 | -0.651 | down |
| MSTRG.7846 | 44 | 55 | 150 | 31 | 42 | 52 | 0.63 | 0.55 | 1.16 | 0.39 | 0.45 | 0.31 | 0.029 | -0.895 | down |
| 6430548M08Rik | 419 | 562 | 1086 | 367 | 439 | 654 | 1.86 | 1.75 | 2.67 | 1.46 | 1.50 | 1.23 | 0.020 | -0.478 | down |
| Klhl3 | 59 | 82 | 105 | 82 | 120 | 243 | 0.20 | 0.19 | 0.19 | 0.25 | 0.30 | 0.34 | 0.008 | 0.703 | up |
| Arhgap6 | 433 | 653 | 1017 | 609 | 676 | 1443 | 2.58 | 2.70 | 3.27 | 3.25 | 3.04 | 3.63 | 0.009 | 0.293 | up |
| Bcl2l14 | 12 | 29 | 87 | 55 | 59 | 258 | 0.12 | 0.29 | 0.72 | 0.75 | 0.67 | 1.64 | 0.014 | 1.463 | up |
| Ltb | 71 | 133 | 351 | 49 | 75 | 168 | 1.46 | 2.27 | 3.92 | 0.91 | 1.16 | 1.45 | 0.021 | -0.920 | down |
| Sh3bp5 | 1631 | 2301 | 4605 | 1425 | 1770 | 3650 | 14.78 | 14.37 | 22.47 | 11.51 | 12.00 | 13.85 | 0.011 | -0.365 | down |
| Snx29 | 223 | 322 | 436 | 194 | 207 | 408 | 2.14 | 2.03 | 2.16 | 1.61 | 1.44 | 1.57 | 0.043 | -0.370 | down |
| Ifnlr1 | 66 | 91 | 473 | 32 | 44 | 176 | 0.38 | 0.36 | 1.47 | 0.16 | 0.19 | 0.42 | 0.036 | -1.347 | down |
| Aebp1 | 153 | 223 | 303 | 216 | 237 | 620 | 1.00 | 0.98 | 1.07 | 1.25 | 1.16 | 1.63 | 0.019 | 0.496 | up |
| Edil3 | 353 | 539 | 875 | 667 | 689 | 1845 | 1.63 | 1.73 | 2.27 | 2.88 | 2.42 | 3.64 | 0.000 | 0.736 | up |
| Gnal | 46 | 111 | 222 | 30 | 67 | 90 | 0.19 | 0.32 | 0.51 | 0.19 | 0.21 | 0.26 | 0.032 | -0.959 | down |
| S100a11 | 11620 | 16858 | 21225 | 14329 | 18249 | 41143 | 548.76 | 549.76 | 539.29 | 605.72 | 646.48 | 809.34 | 0.030 | 0.394 | up |
| S100a13 | 512 | 698 | 1165 | 678 | 812 | 2027 | 17.27 | 16.25 | 21.15 | 20.48 | 20.53 | 28.49 | 0.008 | 0.428 | up |
| Pla1a | 1462 | 1984 | 4041 | 2057 | 2447 | 5785 | 17.87 | 16.75 | 26.57 | 22.51 | 22.44 | 29.47 | 0.021 | 0.379 | up |
| Zfp397 | 601 | 795 | 1680 | 573 | 604 | 1034 | 3.41 | 2.94 | 4.78 | 2.51 | 2.56 | 2.56 | 0.027 | -0.448 | down |
| Megf9 | 360 | 536 | 1161 | 344 | 355 | 716 | 1.10 | 1.13 | 1.92 | 0.94 | 0.82 | 0.91 | 0.012 | -0.526 | down |
| Selenow | 5518 | 8793 | 11181 | 7265 | 9879 | 22687 | 180.91 | 199.09 | 197.24 | 213.22 | 242.98 | 309.85 | 0.013 | 0.473 | up |
| Selenom | 313 | 462 | 498 | 468 | 540 | 949 | 10.63 | 10.72 | 8.95 | 14.07 | 13.56 | 13.35 | 0.040 | 0.503 | up |
| Ndor1 | 167 | 245 | 705 | 29 | 4 | 24 | 1.97 | 1.99 | 4.47 | 0.30 | 0.03 | 0.11 | 0.000 | -4.078 | down |
| Lpar6 | 435 | 587 | 991 | 603 | 621 | 1391 | 1.94 | 1.81 | 2.38 | 2.41 | 2.08 | 2.59 | 0.022 | 0.293 | up |
| Ints6l | 804 | 1010 | 1844 | 730 | 827 | 1497 | 4.39 | 3.67 | 4.88 | 3.38 | 3.20 | 3.20 | 0.033 | -0.301 | down |
| Zfp646 | 1289 | 1848 | 2655 | 1139 | 1385 | 2178 | 5.48 | 5.35 | 5.90 | 4.07 | 4.20 | 3.76 | 0.046 | -0.345 | down |
| Insl6 | 1734 | 2507 | 3465 | 2099 | 2709 | 8225 | 59.10 | 59.01 | 63.54 | 64.04 | 69.25 | 116.77 | 0.046 | 0.519 | up |
| Adgrg3 | 422 | 672 | 1143 | 248 | 316 | 824 | 4.20 | 4.61 | 6.17 | 2.19 | 2.39 | 3.42 | 0.000 | -0.816 | down |
| Nts | 462 | 724 | 1258 | 1015 | 1103 | 7248 | 8.93 | 9.67 | 13.08 | 17.56 | 15.99 | 58.38 | 0.002 | 1.589 | up |
| Spred3 | 138 | 196 | 284 | 203 | 194 | 552 | 0.99 | 0.86 | 0.93 | 1.27 | 0.95 | 1.46 | 0.040 | 0.463 | up |
| Mtr | 534 | 740 | 1568 | 511 | 578 | 1060 | 1.56 | 1.50 | 2.52 | 1.35 | 1.28 | 1.32 | 0.029 | -0.396 | down |
| Sell | 399 | 505 | 727 | 300 | 361 | 485 | 3.37 | 3.11 | 3.18 | 2.12 | 2.12 | 1.90 | 0.036 | -0.536 | down |
| Selp | 79 | 77 | 222 | 162 | 184 | 394 | 0.55 | 0.37 | 0.84 | 1.02 | 0.97 | 1.15 | 0.001 | 0.940 | up |
| Trpm2 | 302 | 446 | 1231 | 238 | 273 | 314 | 1.07 | 1.21 | 2.61 | 0.73 | 0.72 | 0.45 | 0.005 | -1.069 | down |
| Rab27a | 280 | 363 | 1086 | 235 | 251 | 530 | 2.23 | 2.00 | 4.65 | 1.65 | 1.52 | 1.76 | 0.020 | -0.718 | down |
| Etfbkmt | 315 | 370 | 652 | 434 | 486 | 1098 | 5.41 | 4.11 | 5.88 | 6.56 | 5.90 | 7.76 | 0.002 | 0.481 | up |
| Gimap9 | 79 | 98 | 235 | 59 | 62 | 152 | 1.37 | 1.25 | 2.32 | 0.91 | 0.80 | 1.21 | 0.024 | -0.642 | down |
| Dgat2 | 282 | 430 | 1062 | 201 | 248 | 692 | 3.01 | 3.19 | 6.11 | 1.93 | 1.98 | 3.09 | 0.021 | -0.702 | down |
| Csrp1 | 2480 | 3537 | 5257 | 3359 | 3933 | 8644 | 32.82 | 32.33 | 37.44 | 39.79 | 39.05 | 47.66 | 0.001 | 0.380 | up |
| Procr | 24156 | 34386 | 61635 | 32963 | 40263 | 238992 | 365.42 | 359.40 | 502.80 | 446.05 | 457.12 | 1508.11 | 0.036 | 1.026 | up |
| Padi4 | 161 | 201 | 232 | 91 | 113 | 86 | 1.68 | 1.43 | 1.38 | 0.89 | 0.89 | 0.39 | 0.038 | -0.995 | down |
| Cltb | 445 | 697 | 933 | 591 | 785 | 1374 | 4.92 | 5.31 | 5.55 | 5.84 | 6.50 | 6.33 | 0.046 | 0.322 | up |
| Loxl3 | 631 | 946 | 1786 | 955 | 1276 | 3730 | 3.89 | 4.02 | 6.28 | 5.26 | 6.05 | 9.95 | 0.010 | 0.669 | up |
| F8 | 19 | 42 | 69 | 54 | 49 | 110 | 0.05 | 0.07 | 0.16 | 0.12 | 0.09 | 0.11 | 0.046 | 0.699 | up |
| Trem2 | 3121 | 4903 | 5911 | 4371 | 5628 | 10274 | 69.95 | 75.65 | 71.40 | 87.51 | 94.47 | 95.90 | 0.016 | 0.427 | up |
| Myo1d | 240 | 325 | 461 | 156 | 218 | 285 | 1.08 | 1.01 | 1.12 | 0.63 | 0.73 | 0.53 | 0.008 | -0.674 | down |
| Lcn2 | 32898 | 50505 | 113280 | 27198 | 33196 | 85597 | 863.41 | 915.36 | 1536.39 | 637.27 | 653.60 | 896.58 | 0.038 | -0.494 | down |
| Irf5 | 6738 | 9821 | 13656 | 8160 | 9919 | 22197 | 74.38 | 74.76 | 82.59 | 80.70 | 82.20 | 102.91 | 0.049 | 0.272 | up |
| Irf4 | 1696 | 2564 | 6549 | 3272 | 3662 | 9680 | 8.67 | 9.31 | 18.21 | 14.99 | 14.55 | 20.95 | 0.032 | 0.591 | up |
| Cd48 | 1655 | 2333 | 4011 | 2295 | 2674 | 4935 | 33.66 | 32.77 | 43.95 | 42.02 | 40.82 | 41.82 | 0.010 | 0.266 | up |
| Hp | 5551 | 8074 | 15049 | 4452 | 5512 | 7703 | 99.83 | 100.22 | 145.28 | 71.67 | 74.48 | 57.65 | 0.002 | -0.653 | down |
| BC029722 | 576 | 902 | 1475 | 873 | 968 | 1829 | 8.60 | 9.30 | 11.85 | 11.66 | 10.84 | 11.38 | 0.026 | 0.278 | up |
| Stxbp3 | 6419 | 8841 | 12073 | 8266 | 8860 | 19650 | 64.65 | 61.68 | 65.76 | 74.43 | 66.86 | 82.70 | 0.049 | 0.295 | up |
| Stxbp5 | 2377 | 3359 | 5126 | 2228 | 2523 | 4258 | 9.00 | 8.73 | 10.04 | 7.09 | 6.79 | 6.98 | 0.037 | -0.310 | down |
| Il33 | 88 | 98 | 271 | 160 | 207 | 1086 | 0.83 | 0.64 | 1.37 | 1.35 | 1.47 | 4.29 | 0.007 | 1.400 | up |
| Syn1 | 103 | 135 | 215 | 151 | 206 | 385 | 0.76 | 0.68 | 0.87 | 0.99 | 1.14 | 1.19 | 0.001 | 0.619 | up |
| Plat | 113 | 162 | 480 | 297 | 361 | 1019 | 1.11 | 1.07 | 2.52 | 2.57 | 2.63 | 4.11 | 0.002 | 1.112 | up |
| Kiz | 379 | 613 | 1008 | 374 | 400 | 951 | 4.22 | 4.68 | 6.02 | 3.70 | 3.31 | 4.38 | 0.050 | -0.295 | down |
| Kit | 689 | 1023 | 2487 | 629 | 671 | 1505 | 3.17 | 3.25 | 6.16 | 2.61 | 2.31 | 2.89 | 0.017 | -0.573 | down |
| Nhsl2 | 81 | 145 | 304 | 77 | 57 | 184 | 0.24 | 0.24 | 0.31 | 0.13 | 0.08 | 0.22 | 0.026 | -0.768 | down |
| MSTRG.21378 | 421 | 568 | 940 | 559 | 636 | 1249 | 8.12 | 7.57 | 9.76 | 9.65 | 9.20 | 10.04 | 0.019 | 0.271 | up |
| Dcaf17 | 385 | 500 | 1010 | 359 | 404 | 748 | 1.47 | 1.41 | 2.03 | 1.18 | 1.19 | 1.13 | 0.036 | -0.345 | down |
| H2-Bl | 141 | 264 | 176 | 190 | 615 | 725 | 2.97 | 3.86 | 2.00 | 3.59 | 9.78 | 6.40 | 0.021 | 1.203 | up |

**Key resource table**

| Reagent or resource | Source | Identifier |
| --- | --- | --- |
| Antibody |  |  |
| PE anti-mouse CD4 Antibody | Biolegend | Cat# 100408 |
| PerCP/Cyanine5.5 anti-mouse CD3 Antibody | Biolegend | Cat# 100218 |
| APC anti-mouse CD8a Antibody | Biolegend | Cat# 100708 |
| PE anti-mouse IFN-γ Antibody | Biolegend | Cat# 505808 |
| FITC anti-mouse TNF-α Antibody | Biolegend | Cat# 506304 |
| PE anti-mouse Perforin Antibody | Biolegend | Cat# 154306 |
| APC anti-mouse CD11c Antibody | Biolegend | Cat# 117310 |
| PerCP/Cyanine5.5 anti-mouse CD11b Antibody | Biolegend | Cat# 101228 |
| FITC anti-mouse CD103 Antibody | Biolegend | Cat# 121420 |
| Granzyme B Monoclonal Antibody, PE | eBioscienc | Cat# 12-8898-82 |
| MHC Class II Monoclonal Antibody, PE | eBioscienc | Cat# 12-5321 |
| FOXP3 Antibody, PerCP-Cyanine5.5, | eBioscienc | Cat# 45-5773-82 |
| CD25 Monoclonal Antibody, APC, | eBioscienc | Cat# 17-0251-82 |
| FITC anti-mouse CD45 Antibody | Biolegend | Cat# 103108 |
| APC anti-mouse CD45 Antibody | Biolegend | Cat# 103112 |
| APC anti-mouse Ki-67 Antibody | Biolegend | Cat# 652406 |
| APC anti-mouse CD366 (Tim-3) Antibody | Biolegend | Cat# 134007 |
| PE anti-mouse CD80 Antibody | Biolegend | Cat# 104707 |
| PE/Cyanine7 anti-mouse CD86 Antibody | Biolegend | Cat# 105014 |
| FITC anti-mouse CD206 (MMR) Antibody | Biolegend | Cat# 141704 |
| PE anti-mouse Ly-6G/Ly-6C (Gr-1) Antibody | Biolegend | Cat# 108408 |
| PE anti-mouse Siglec H Antibody | Biolegend | Cat# 129606 |
| FITC anti-mouse CD45R/B220 Antibody | Biolegend | Cat# 103205 |
| PE anti-mouse H-2Kb Antibody | Biolegend | Cat# 116508 |
| PE anti-mouse CD49b (pan-NK cells) Antibody | Biolegend | Cat# 108908 |
| APC anti-mouse CD124 (IL-4Rα) Antibody | Biolegend | Cat# 144807 |
| FITC anti-mouse F4/80 Recombinant Antibody | Biolegend | Cat# 157309 |
| PE-anti-SIINFEKL-Tetramer | MLB | Cat#TS-5001-1C |
| Phospho-TBK1/NAK | Cell signaling | Cat# 5483S |
| Anti-human/mouse TBK1/NAK Rb mAb | Cell signaling | Cat# 3504S |
| Anti-human/mouse phospho IRF3 | Cell signaling | Cat# 29047S |
| Anti-human/mouse IRF3 Rb mAb | Cell signaling | Cat# 4302S |
| Anti-STING Rabbit mAb #13647 | Cell signaling | Cat# 13647s |
| Phospho-STING Rabbit mAb | Cell signaling | Cat# 19781s |
| Anti-mouse β-Actin Antibody | Santa Cruz Biotechnology | Cat# sc-47778 |
| Recombinant Anti-CD4 antibody | Abcam | Cat# ab133616 |
| Anti-CD1c antibody | Abcam | Cat# ab156708 |
| anti-mouse CD11c Antibody | Abcam | Cat# 563978 |
| anti-mouse FOXP3 Antibody | Abcam | Cat# 563978 |
| Anti-TIM 3 antibody | Abcam | Cat# ab185703 |
| InVivoMAb anti-mouse TIM-3 (CD366) | BioXcell | Cat# BE0115 |
| InVivoPlus anti-mouse CD4 | BioXcell | Cat# BP0003 |
| InVivoPlus anti-mouse CD8α | BioXcell | Cat# BP0004 |
| Anti-Asialo-GM1 | Biolegend | Cat#146002 |
| Chemicals, peptides, and proteins |  |  |
| ADU-S100 | MCE | HY-12885B |
| Coldronate liposome | LIPOSOMA | Cat# C010 |
| Staining buffer | Invitrogen | Cat# 2231158 |
| Collagenase A | Millipore Sigma | 11088793001 |
| DNase I, grade II from bovine pancreas | Roche | 10104159001 |
| Zombie NIR™ Fixable Viability Kit | Biolegend | Cat# 423106 |
| Recombinant mouse GM-CSF | Peprotech | Cat# 315-03 |
| Recombinant mouse IL-4 | Peprotech | Cat# 214-14 |
| OVA Peptide 323-339 | MCE | HY-P0286 |
| OVA Peptide 257-264 | MCE | HY-P1489 |
| TNF-alpha/TNFSF2 protein, Mouse | MCE | HY-P70571 |
| IL-2 Protein, Mouse | MCE | HY-P7077 |
| M-CSF Protein, Mouse | MCE | HY-P7085 |
| TBST, 20× | Solarbio | Cat# T1082 |
| Protein Ladder | ThermoFisher | Cat# 26616 |
| Trizol | ThermoFisher | 15596026 |
| Critical commercial assays |  |  |
| CD4^+^ T Cell Isolation Kit, mouse | Miltenyi | 130-090-860 |
| CD11c MicroBeads UltraPure, mouse | Miltenyi | 130-125-835 |
| CD8a^+^ T Cell Isolation Kit, mouse | Miltenyi | 130-090-859 |
| Pan T Cell Isolation Kit II, mouse | Miltenyi | 130-095-130 |
| NK Cell Isolation Kit, mouse | Miltenyi | 130-115-818 |
| Mouse Th1/Th2/Th17 Cytokine Kit | BD | Cat# C0051 |
| CFSE Cell Proliferation Assay and Tracking Kit | Beyotime | Cat# 563978 |
| 2X SG Fast qPCR Master Mix (Low Rox) | Sangon | B639272-0005 |
| Opal 7-color Manual IHC Kit | PerkinElmer | NEL811001KT |
| Foxp3/Transcription Factor Staining Buffer Set | Invitrogen | 00-5523-00 |
| LEGEND MAX™ Mouse IFN-γ ELISA Kit | Biolegend | Cat# 430807 |
| Cell activation cocktail (with Brefeldin A) | Biolegend | Cat# 423304 |
| Mouse CXCL9/MIG Quantikine ELISA Kit | R&D Systems | Cat# DY492-05 |
| Mouse CXCL10/IP-10/CRG-2 DuoSet ELISA | R&D Systems | Cat# DY466-05 |
| Mouse TNF-alpha DuoSet ELISA | R&D Systems | Cat# DY410-05 |
| ELISA Kit for Aspartate Aminotransferase | Cloud-Clone | SEB214Mu |
| ELISA Kit for Alanine Aminotransferase | Cloud-Clone | SEA207Mu |
| Mouse IFN-γ Precoated ELISpot Kit (Strips) | Dakewe Biotech | Cat# 2210005 |
| CytoTox 96® Non-Radioactive Cytotoxicity Assay | Progema | REF#1781 |
